# *Robinia pseudoacacia* L. Flower Exosome‐Like Nanoparticles (RFELNs) Activate AhR/IL‐22 to Relieve Intestinal Barrier Dysfunction Through Regulating Gut Microbiota‐Interrelated Tryptophan Metabolism in Ulcerative Colitis Mice

**DOI:** 10.1155/mi/1794173

**Published:** 2026-01-22

**Authors:** Feihan Shen, Kewen Sun, Jianguo Song, Xueping Chen, Juan Dai, Ying Qi, Liwen Zhang, Liang Ma

**Affiliations:** ^1^ Department of Gastroenterology, The First People’s Hospital of Changzhou, The Third Affiliated Hospital of Soochow University, Changzhou, Jiangsu, China, suda.edu.cn; ^2^ Department of Gastroenterology, The Fifth People’s Hospital of Xinjiang Uygur Autonomous Region, Xin Jiang, China; ^3^ Department of Gastroenterology, The People’s Hospital of Wuqia, Xin Jiang, China; ^4^ Department of Pediatrics, the Second People’s Hospital of Changzhou, The Third Affiliate Hospital of NanJing medical University, Changzhou, Jiangsu, China, njmu.edu.cn

**Keywords:** AhR, IL-22, ILC3, RFELNs, tryptophan metabolism, UC

## Abstract

**Background:**

Intestinal barrier dysfunction is a key driver of ulcerative colitis (UC) recurrence and chronic persistence. Modulating group 3 innate lymphoid cells (ILC3) activity and tryptophan‐derived metabolites is crucial for enhancing mucosal repair in UC. *Robinia pseudoacacia* L. flower exosome‐like nanoparticles (RFELNs) could ameliorate intestinal mucosal injury in mice. This study aimed to investigate the impact of RFELNs on intestinal barrier repair in UC mice and explore the underlying mechanisms.

**Methods:**

Changes in body weight, food intake, DAI score, colon length, pathological score, and inflammatory factor level were performed to assess the therapeutic effect of RFELNs on DSS‐stimulated UC mouse models. The effects of RFELNs on intestinal barrier integrity were assessed by intestinal barrier permeability analysis, Alcian Blue staining, immunohistochemistry (IHC), and western blot assays. IL‐22 level was measured by immunofluorescent staining and ELISA assay. Besides, flow cytometry was performed to detect the proportions of ILC3 and NCR^+^ILC3 in the colon. Subsequently, an in vitro culture system consisting of NCM460 cells and MNK3 cells was established to determine potential mechanism of RFELNs’ influence on UC.

**Results:**

RFELNs prominently relieved pathological symptoms in UC mice, including weight loss, enhanced DAI score, shortened colon, and pathological colon damage. Moreover, RFELNs decreased the concentration of FITC‐dextran and DAO level and enhanced D‐lactate levels. Additionally, RFELNs significantly enhanced the number of colonic goblet cells, restored epithelial tight junctions (TJs), and upregulated TJ protein levels. Moreover, RFELNs enhanced IL‐22 expression and the proportion of ILC3 cells and NCR^+^ILC3 cells. The protective effect of RFELNs on UC depends on AhR. Further, RFELNs activated AhR pathway by increasing the content of indole derivatives produced by tryptophan metabolism, thus promoting the repair of intestinal barrier damage.

**Conclusion:**

RFELNs restored intestinal barrier function in UC mice by activating AhR/IL‐22 signaling through regulation of gut microbiota‐dependent tryptophan metabolism.

## 1. Introduction

Ulcerative colitis (UC) is a chronic inflammatory bowel disease with multifactorial etiology and has emerged as a significant global health burden [1]. Over recent decades, the global incidence of UC has risen substantially, showing a sustained annual increase [2, 3]. UC lesions predominantly involve the mucosa and submucosa, most commonly affecting the rectum and sigmoid colon, but may extend to the descending colon or involve the entire colon [4]. The primary symptoms are abdominal pain, diarrhea, and bloody stool, with up to 15% of patients experiencing severe symptoms at their initial visit [5–6, 7]. The prevailing view on UC pathogenesis is that a combination of environmental and host factors increases susceptibility, with epithelial barrier disruption, intestinal flora imbalance, and immune dysregulation playing key roles in triggering and sustaining inflammation [7, 7]. Therefore, protecting the intestinal barrier to prevent pathogenic invasion and suppressing inflammation are effective strategies for treating UC.

Group 3 innate lymphoid cells (ILC3s) play a crucial role in maintaining barrier tissue equilibrium and are essential for regulating the symbiotic relationship between the host and commensal organisms. These cells quickly react to tissue injury, inflammatory signals, and pathogenic threats to facilitate the recovery of tissue integrity [8, 8]. Under normal conditions, ILC3s stimulate intestinal epithelial cells to produce antimicrobial peptides (AMPs) by secreting IL‐22 [9, 10]. This regulates symbiotic bacteria growth and enhances probiotic colonization by inducing fucosylation of epithelial cell surface proteins, thus maintaining intestinal immune tolerance and barrier function [11]. Besides, IL‐22 promotes goblet cell proliferation, facilitates intestinal mucus layer formation, and enhances tight junction (TJ) protein expression in epithelial cells, thereby providing multi‐faceted protection to the intestinal mucosa [12]. Transcription factor AHR is crucial for ILC3 survival and function [13]. AHR signaling activation enhances CYP1A1 expression, leading to increased IL‐22 secretion [14]. AhR ligands include aromatic hydrocarbons from external sources as well as endogenous compounds like indole derivatives from tryptophan catabolism [15]. Wang et al. [16] indicated that Gegen Qinlian decoction could repair intestinal barrier in UC mice, but this protective effect was abolished by AhR antagonist (CH223191). Therefore, activating the AHR signaling to enhance IL‐22 secretion by ILC3 is vital for maintaining intestinal epithelial homeostasis.

Gut microbiota dysbiosis disrupts immune regulation and epithelial function through aberrant pro‐inflammatory mediator production and altered microbial metabolites [17, 18]. This cascade compromises the intestinal epithelial barrier, leading to mucosal dysfunction, chronic inflammation, and ultimately UC pathogenesis [19]. Proteobacteria include various pathogenic bacteria, such as Enterohepatic and Helicobacter, which are linked to intestinal mucosal injury and inflammatory diseases [20]. UC patients exhibit gut microbiota dysbiosis characterized by reduced Firmicutes and Bacteroidetes abundances alongside increased Proteobacteria, which collectively represent the three dominant bacterial phyla in the human gut [21]. Meanwhile, significant microbiota alterations were observed in UC mice: decreased abundances of Lactobacillus (Lactobacillaceae), Ruminococcus, and other Lactobacillales taxa, but increased Prevotella, Fusobacterium, and Escherichia‐Shigella [18]. Metabolites of tryptophan, including indole‐propionic acid (IPA), indole‐3‐lactic acid (ILA), and indole‐acetic acid (IAA), produced by intestinal microbiota function as AHR activators. These compounds promote IL‐22 production by ILC3s, facilitating the restoration of damaged intestinal epithelium [22]. Fecal microbiota transplantation (FMT) could treat UC by restoring the composition and function of the gut bacterial community [23]. Therefore, the regulation of gut microbiota and its metabolic pathway is also one of the important ways for the repair of UC intestinal mucosal damage.

*Robinia pseudoacacia* L. flowers, a traditional medicinal material, are also used as a functional food [24]. *Robinia pseudoacacia* L. flowers contain various flavonoids, including hyperoside, rutin, and quercetin, and demonstrate a broad range of pharmacological effects, such as hemostatic, antibacterial, and antioxidant activities. Plant exosome‐like nanoparticles (PELNs), released by various plants, have immunomodulatory properties, enhance the intestinal microbiota, and support interspecies communication [25, 26]. PELNs provide several benefits over other approaches, including low toxicity, efficient cellular uptake, reduced immunogenicity, and high biocompatibility and stability [27]. These qualities make PELNs highly promising for clinical use in treating gastrointestinal diseases. For instance, ELNs from *Portulaca oleracea* L. relieved DSS‐caused colitis by promoting positive CD4(+)CD8(+)T cell expansion [28]. ELNs from mulberry bark impeded DSS‐triggered colitis by regulating AhR/COPS8 signaling [29]. ELNs derived from ginseng improved the progression of colitis by inhibiting inflammatory cytokines [30]. Moreover, given the reported pharmacological effects of exosome‐like nanoparticles derived from *Robinia pseudoacacia* flowers (RFELNs), including the amelioration of intestinal mucosal injury and the restoration of gut microbiota dysbiosis [31], we hypothesized that RFELNs may possess therapeutic potential for mucosal damage associated with UC.

In this study, we demonstrated for the first time that RFELNs can alleviate intestinal inflammation and enhance intestinal barrier function in a mouse model of UC. Mechanistically, RFELNs exert their protective effects by activating the AhR/IL‐22 signaling pathway in ILC3s, and this effect is dependent on AhR. These findings provide new insights into the therapeutic potential of RFELNs for UC treatment.

## 2. Methods

### 2.1. Isolation and Identification of RFELNs

*Robinia pseudoacacia* L. flowers were obtained from Shanghai, China. RFELNs were obtained through ultracentrifugation. In short, *Robinia pseudoacacia* L. flowers were cut, washed, and dried. Subsequently, the sample was chopped, soaked in 75% alcohol for 1 min, washed with distilled water to remove alcohol residue, dried, added 20 mL enzyme solution, enzymatic hydrolysis at 50°C for 6 h. The sample is then centrifuged and the supernatant is carefully transferred to a new tube and centrifuged again to remove the larger vesicles and dead cells in the supernatant. Then, the supernatant was filtered by 0.45 μm membrane at 4°C, 100,000×*g*, and ultracentrifugation twice for 70 min. The precipitate was re‐suspended in 150 μL PBS. 20 μL of RFELNs was observed by transmission electron microscope (TEM), 10 μL was determined by particle size, and the rest was stored at −80°C. The size was assessed via dynamic light scattering (DLS) using a Zetasizer Nano ZS (Malvern Instrument, UK).

### 2.2. Animals

Male C57BL/6J mice (7–8 weeks, 20–24 g) were obtained from GemPharmatech Co., Ltd. (Foshan, Guangdong, China). The experimental mice were kept in standard enclosures within a climate‐controlled environment (23 ± 2°C) under a 12‐h light/dark cycle, with unlimited access to water and food. Approval for all animal studies was granted by the Ethics Committee of The First People’s Hospital of Changzhou (2023 CZYY20222‐60).

After 1 week of adaptive feeding, the mice were randomly divided into four groups: sham, DSS, DSS + RFELNs, and DSS + RFELNs + CH223191 groups. Except for the sham group, the other mice were given 2.5% DSS solution to induce UC for seven consecutive days. In DSS + RFELNs group, UC mice were gavaged with 100 μL PBS with dissolved 5 × 10^5^ particles of RFELNs for 5 days. The dose of RFELNs (5 × 10^5^ particles/mouse) was determined based on our preliminary dose–response experiments and relevant studies on plant‐derived exosome‐like nanoparticles, which showed significant biological effects in similar dosage ranges [31]. For DSS + RFELNs+CH223191 group, UC mice were intraperitoneally injected with CH223191 (10 mg/kg) first and gavaged with 100 μL PBS with dissolved 5 × 10^5^ particles of RFELNs after 30 min for 5 days. The dose of CH223191 (10 mg/kg) was selected according to previous studies demonstrating effective AhR inhibition in mouse models [16].

The mice were monitored daily, and their disease activity index (DAI), including body weight loss and visible blood, was detected as previously described [32]. On day 12, mice were humanely euthanized by an overdose of sodium pentobarbital (150 mg/kg, intraperitoneally), followed by cervical dislocation. Blood samples were collected, and serum was stored at −80°C for further analysis. The isolated colon was harvested for pathological examination.

### 2.3. Intestinal Barrier Permeability Analysis

After overnight fasting, the mice were orally administered fluorescein isothiocyanate (FITC)‐dextran (MW: 4 kD) 4 h before sacrifice. Blood samples (0.1 mL) were obtained from the mice, and fluorescence intensity at 485 nm was detected using a MicroPlate Reader (SpectraMax iD5). The concentrations of d‐lactic acid (D‐LA) and diamine oxidase (DAO) were measured using ELISA kits.

### 2.4. Western Blotting Analysis

Protein samples from colon tissues and cultured cells were extracted using RIPA‐lysis buffer. Proteins from cells were extracted using a nuclear and cytoplasmic protein extraction kit (Beyotime, China). 30 μg of extracted protein were separated by SDS‐PAGE and transferred to a PVDF membrane. The membrane was then blocked for 2 h at room temperature with 5% skim milk powder. Then, primary antibodies against ZO‐1, Occludin, CYP1A1, AhR, GAPDH, Lamin B, and β‐actin were added, and the membranes were incubated overnight at 4°C. On the second day, matched secondary antibodies were added and incubated. The membrane was treated with ECLPlus kit and chemiluminescence imager.

### 2.5. Quantitative Real‐Time Polymerase Chain Reaction (PCR)

Total RNAs were extracted using TRIzol Reagent (Invitrogen). cDNA was synthesized using the High‐Capacity cDNA Reverse Transcription Kit (Applied Biosystems). qRT‐PCR was performed on the 480 II Real‐Time PCR System using the ChamQ Universal SYBR qPCR Master Mix (Vazyme). Target gene expression was normalized to GAPDH levels, and relative expression was determined using the 2^−ΔΔCt^ method.

### 2.6. Immunohistochemistry (IHC)

Colonic tissue specimens preserved in paraffin were initially processed through xylene dewaxing and alcohol gradient rehydration. Antigenic epitopes were subsequently exposed through citrate buffer treatment. To eliminate endogenous peroxidase interference, samples were exposed to 0.3% H_2_O_2_ solution, followed by 60‐min blocking with 5% normal goat serum. Primary antibody incubation was performed at 4°C for 16 h targeting TJ proteins (ZO‐1, Occludin) and the intestinal mucin marker (MUC2). Following extensive PBS washes (≥5 cycles), specimens were treated with species‐specific secondary antibodies for half an hour. Chromogenic visualization was achieved through diaminobenzidine (DAB) substrate reaction, with hematoxylin providing nuclear counterstaining. Finally, processed sections were dehydrated, permanently mounted, and analyzed using an Olympus BX46 brightfield microscope system.

### 2.7. Immunofluorescent Staining

Colon tissues were encased in paraffin, sectioned, degreased with ether, and dehydrated through an alcohol gradient. The sections are heated in a sodium citrate buffer to promote antigen recovery. Sections were sealed with donkey serum for 1 h, washed three times with PBS, and incubated overnight with the first antibody of IL‐22 (1:400, GB11259‐100, Servicebio). After washing three times with PBS, sections were exposed with goat anti‐rabbit IgG for 1 h. After another three PBS washes, they were stained with DAPI and observed with a fluorescence microscope.

### 2.8. H&E Staining

Distal colon tissues were fixed in 4% paraformaldehyde, embedded in paraffin, sectioned at a thickness of 5 μm, and stained with hematoxylin and eosin (H&E). Histological images were captured using a light microscope. The histological score was assessed based on previously established criteria [33], encompassing seven parameters: inflammation (score 0–4), crypt damage severity (0–4), immune cell infiltration (0–3), submucosal edema (0–3), goblet cell depletion (0–3), epithelial hyperplasia (0–3), and presence of crypt abscesses (0–2).

To evaluate mucus‐producing goblet cells, Alcian Blue–Periodic Acid‐Schiff (AB–PAS) staining was performed using a commercial staining kit (Solarbio, Beijing, China) according to the manufacturer’s instructions [34, 35].

### 2.9. ELISA Assay

Mouse IL‐6 and IL‐1β ELISA kits were performed to evaluate cytokine contents in cell culture supernatant.

### 2.10. Cell Culture and Treatments

MNK‐3 and NCM460 cells were obtained from Bluefbio and ATCC Cell Bank (Shanghai, China), and both were cultured in DMEM medium supplemented with 10% FBS and 1% penicillin/streptomycin at 5% CO_2_ and 37°C.

Experiment 1: Four groups of MNK‐3 cells were prepared: Control, and low (5 × 10^3^ particles/mL), medium (2.5 × 10^4^ particles/mL), and high (5 × 10^4^ particles/mL) dose groups of RFELNs. After each dose of RFELNs was incubated with MNK‐3 cells for 24 h, the supernatant was collected, and nuclear and cytoplasmic proteins were extracted, and the related indexes were detected.

Experiment 2: The experiment was divided into the following 4 groups: (1) MNK‐3 cells incubated with culture supernatants of untreated NCM460 cells (control). (2) MNK‐3 cells incubated with culture supernatants of LPS (10 ng/mL)‐treated NCM460 cells (LPS). (3) MNK3 cells were treated RFELNs (5 × 10^4^ particles/mL) and incubated with culture supernatants of LPS (10 ng/mL)‐treated NCM460 cells (LPS + RFELNs). (4) MNK3 cells were treated RFELNs and AhR inhibitor (CH223191, 5 μM) and incubated with culture supernatants of LPS‐treated NCM460 cells (LPS + RFELNs+CH).

### 2.11. CCK‐8 and Treatment

The effect of RFELNs (5 × 10^3^, 2.5 × 10^4^, and 5 × 10^4^ particles/mL) on MNK‐3 cell viability was evaluated using CCK‐8 reagent (APExBIO, USA).

### 2.12. Flow Cytometry Analysis

Lamina propria lymphocytes were isolated from the mouse colon. Cells were stained with fluorochrome‐conjugated antibodies for surface markers to identify ILC3 (CD45^+^) and NCR^+^ILC3 (Nkp46). Stained cells were analyzed using a flow cytometer.

### 2.13. ELISA

The ELISA kit was used to detect the content of IL‐22 in the supernatant of cells.

### 2.14. Transepithelial Electrical Resistance (TEER) Assay and Epithelial Paracellular Permeability

Cells (4 × 10^5^ cells/mL) were seeded into the apical chamber and cultured in the basal chamber for 21 days in 1.0 mL of RPMI‐1640 complete medium. Epithelial resistance was monitored with a Millicell MR‐2 cell resistance meter. Before use, the electrode was sterilized by soaking in 70% ethanol for 15 min, air‐dried, and equilibrated in a sterile electrolyte similar to the culture medium. To measure resistance, the electrode ends were immersed in the medium of the transwell lower chamber. Resistance values were recorded after measuring two blank controls. A TEER value of 200–600 Ώ cm^2^ indicated the establishment of TJs, signaling readiness for drug treatment. Paracellular permeability was assessed using a FITC‐conjugated dextran probe (FD‐4). After 24 h of LPS and/or RFELNs treatment, 1 mg/mL FD‐4 was added to the apical chamber, PBS to the basolateral chamber, and cells were incubated at 37°C for 1 h. Basolateral absorbance was determined at excitation/emission wavelengths of 490/520 nm using a VersaMax microplate reader.

### 2.15. S rRNA Gene Sequencing

Fecal genomic material was isolated and assessed for integrity through agarose gel separation. Amplification of bacterial phylogenetic markers was achieved through PCR targeting the V3–V4 variable domains of the 16S ribosomal RNA gene. Resultant amplicons were size‐fractionated on 2% agarose matrices, subsequently purified, and subjected to quantitative analysis. Sequencing libraries were constructed using the TruSeq DNA LT Sample Preparation System, followed by high‐throughput sequencing on the Illumina MiSeq system. Microbiome profiling and bioinformatics analysis were conducted by MetaboProfile Biotechnology (Shanghai, China).

### 2.16. Targeted Metabolomics Analysis

Targeted metabolomics analysis of murine fecal samples was performed using a Q300 Kit (MetaboProfile, Shanghai, China). Quantification was carried out with the ACQUITY UPLC‐Xevo TQ‐S system (Waters Corp., Milford, MA, USA) coupled to a tandem mass spectrometer (UPLC‐MS/MS) by MetaboProfile Biotechnology Co., Ltd. (Shanghai, China).

### 2.17. Statistical Analysis

Statistical analyses in this study were performed using SPSS 20.0 software (IBM, USA). All data acquired were expressed as the mean ± standard deviation (SD). The significance between two or more groups was determined by Student’s *t*‐test or one‐way ANOVA followed by Tukey‒Kramer multiple comparisons test. Any difference with *p*  < 0.05 was considered statistically significant.

## 3. Results

### 3.1. Characterization of RFELNs

RFELNs were extracted from fresh *Robinia pseudoacacia* L. flowers using ultracentrifugation, as illustrated in Figure 1A. The morphology of RFELNs was examined by TEM and cryogenic TEM (Cryo‐TEM) (Figure 1B), while their average particle size (~176.3 nm) was determined using DLS (Figure 1C).

Figure 1Characterization of RFELNs. (A) A flowchart of RFELNs isolation by ultracentrifugation. (B) TEM (right) (Scale bar = 100 nm) and Cryo‐TEM (left) (Scale bar = 100 nm) image of RFELNs. (C) Analysis of particle size.

**Figure:**
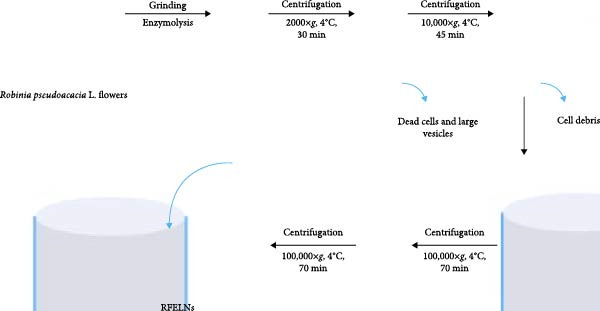
(A)

**Figure:**
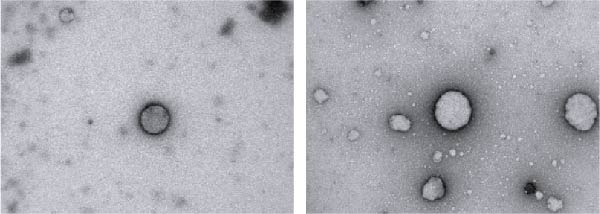
(B)

**Figure:**
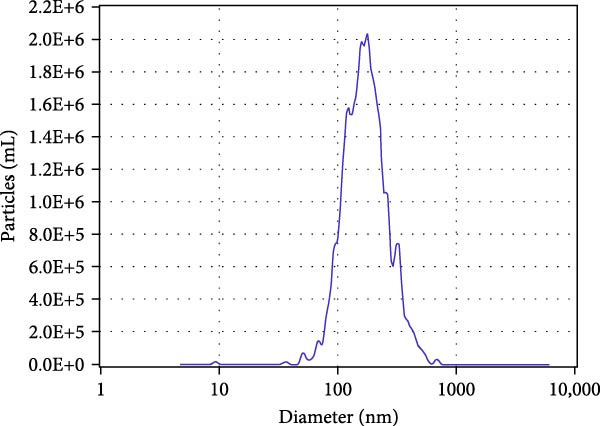
(C)

### 3.2. RFELNs Alleviated Symptoms of UC in Mice

To evaluate the therapeutic effects of RFELNs on UC, RFELNs were administered to DSS‐induced UC mice (Figure 2A). As shown in Figure 2B–D, DSS‐treated mice exhibited typical UC symptoms, including significant weight loss, reduced food intake, and elevated DAI scores. These symptoms were markedly alleviated following RFELNs administration. Furthermore, H&E staining revealed that RFELNs significantly reduced colonic inflammatory cell infiltration, attenuated muscular thickening, preserved glandular architecture, and mitigated epithelial cell shedding (Figure 2E). In addition, the mRNA levels of pro‐inflammatory cytokines TNF‐α, IL‐6, and IL‐1β were markedly upregulated in DSS‐treated mice, whereas RFELNs treatment reversed these changes (Figure 2F). Collectively, these findings suggested that RFELNs effectively alleviate DSS‐induced UC symptoms in mice.

Figure 2RFELNs alleviated symptoms of UC in mice. (A) Study design of mice experiment. (B) Daily changes in body weight in sham, DSS, and RFELNs groups (*n* = 6). (C) Daily changes in food intake in sham, DSS, and RFELNs groups (*n* = 6). (D) Daily changes in disease activity index (DAI) score in sham, UC, and RFELNs groups (*n* = 6). (E) HE staining of colon tissues and histological score (Scale bar = 100 μm). (F) Changes in TNF‐α, IL‐6, and IL‐1β mRNA of the colon (*n* = 6).  ^∗^ *p*  < 0.05,  ^∗∗^ *p*  < 0.01,  ^∗∗∗^ *p*  < 0.001.

**Figure:**
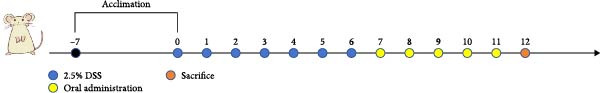
(A)

**Figure:**
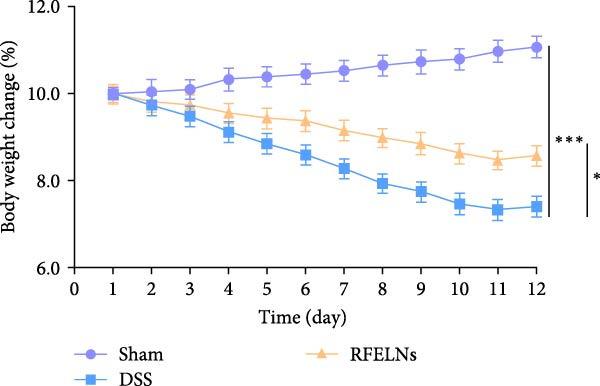
(B)

**Figure:**
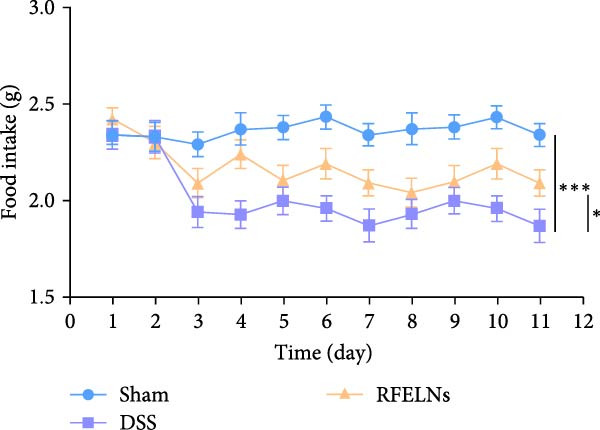
(C)

**Figure:**
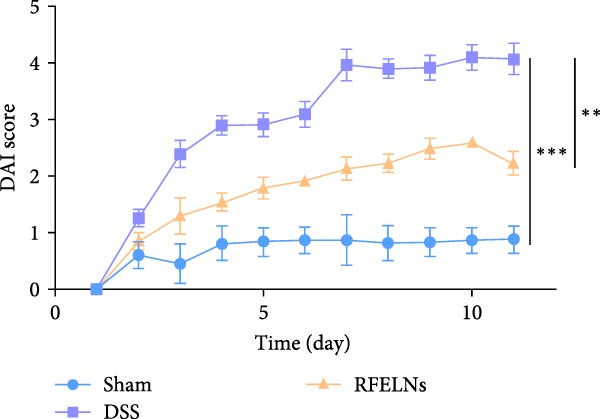
(D)

**Figure:**
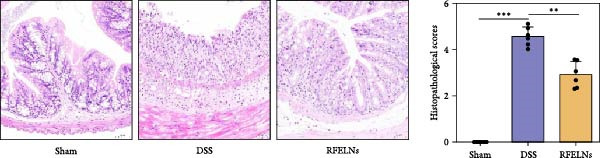
(E)

**Figure:**
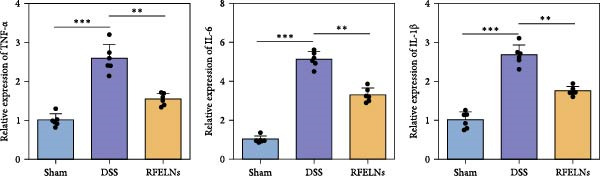
(F)

### 3.3. RFELNs Enhanced Intestinal Barrier Function in UC Mice

Given that intestinal epithelial barrier dysfunction plays a vital role in the pathogenesis of UC [36], we further evaluated the influence of RFELNs on the structure and function of the intestinal barrier in UC mice. The concentration of FITC‐dextran in serum was used to evaluate the intestinal permeability of UC mice. As displayed in Figure 3A, RFELNs treatment reduced the fluorescence intensity of FITC in the serum of DSS‐triggered mice. Moreover, RFELNs reduced the elevated serum DAO levels, a marker of mucosal barrier breakdown, in DSS‐induced mice (Figure 3B). D‐lactate, produced by symbiotic bacteria, promotes epithelial cell regeneration and development [32, 37]. Here, we also found that RFELNs‐supplemented UC mice displayed enhanced d‐lactate levels (Figure 3C). The integrity of the mucosal membrane is largely maintained by mucus‐secreting goblet cells [38]. Colonic tissues from RFELNs‐supplemented mice, stained with Alcian blue, exhibited a higher number of goblet cells (Figure 3D). Mice treated with RFELNs showed higher MUC2 expression compared to mice treated with DSS, indicating mucus preservation after RFELNs treatment. Intestinal TJs dysfunction and abnormal protein expression are associated with epithelial barrier damage [39]. The findings from IHC and western blot assays indicated that the administration of RFELNs could increase the level of ZO‐1 and Occludin in DSS‐stimulated mice (Figure 3E, F).

Figure 3RFELNs enhanced intestinal barrier function in UC mice. Mice were randomly assigned to three groups: sham, DSS, and RFELNs. Changes in intestinal permeability were quantified by assessing the ratio of (A) serum FITC‐glucan 4 kDA, (B) diamine oxidase (DAO), and (C) D‐LA. (D) Staining of Alcian Blue. (E and F) MUC2, ZO‐1, and occludin expressions were measured by western blot and IHC assays. (G) IL‐22 protein expression was measured by immunofluorescence staining in the colon (Scale bar = 100 μm). (H) Change in the proportion of ILC3 and NCR^+^ILC3 cells in the colon.  ^∗^ *p*  < 0.05,  ^∗∗^ *p*  < 0.01,  ^∗∗∗^ *p*  < 0.001.

**Figure:**
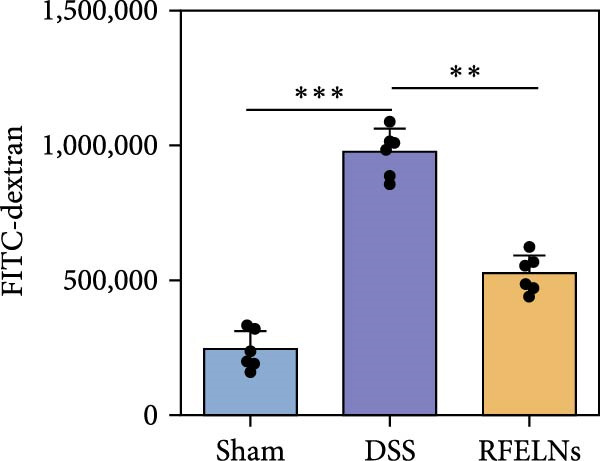
(A)

**Figure:**
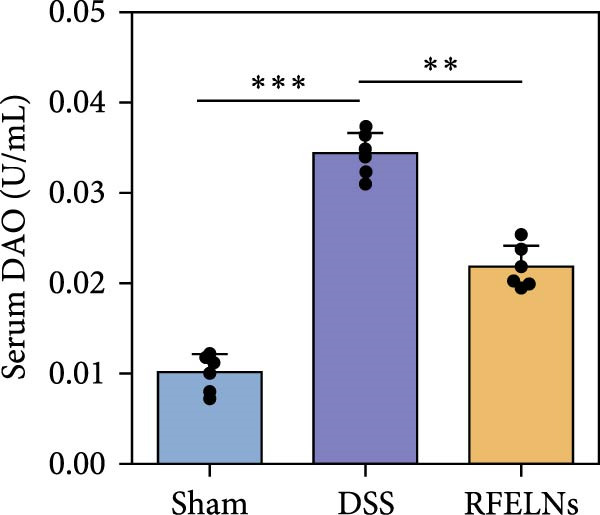
(B)

**Figure:**
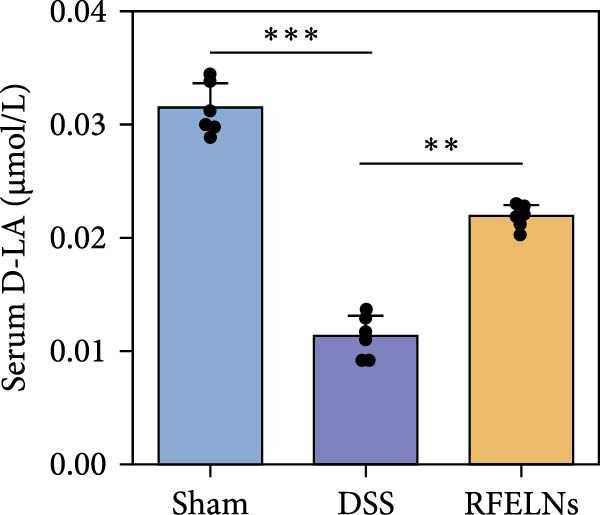
(C)

**Figure:**
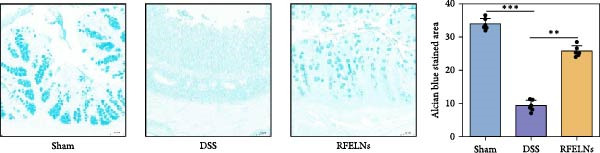
(D)

**Figure:**
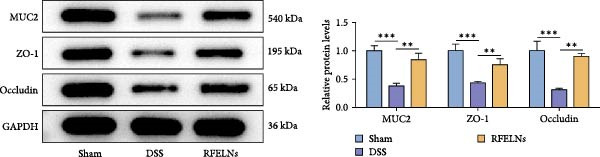
(E)

**Figure:**
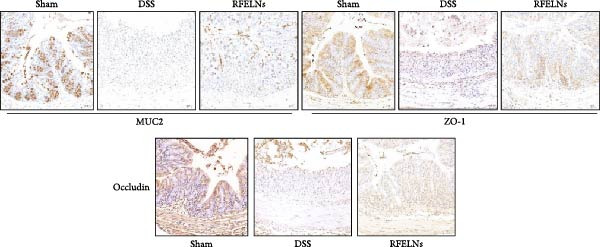
(F)

**Figure:**
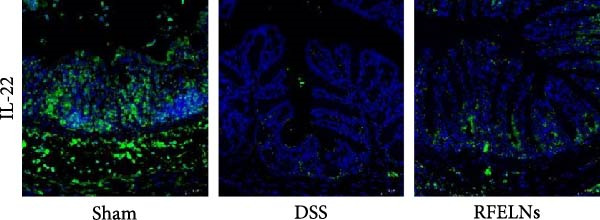
(G)

**Figure:**
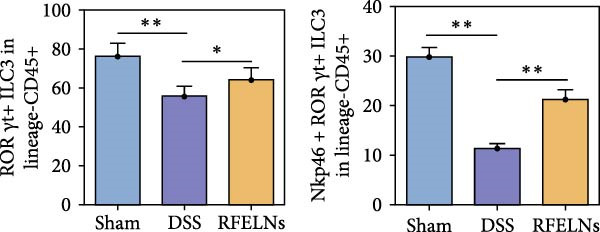
(H)

To elucidate the underlying mechanism by which RFELNs enhance intestinal barrier integrity and strengthen TJs in UC mice, we further assessed the levels of the repair factor IL‐22 in colon. Results of Figure 3G, the fluorescence intensity of IL‐22 in colon induced by DSS was distinctly lower than that in sham group, which was consistent with previous study results [18, 40]. However, treatment with RFELNs can partially restore IL‐22 levels in the colon of mice, suggesting that upregulation of IL‐22 is involved in the remission of intestinal mucosal injury caused by RFELNs. Further, RFELNs administration enhanced the percentage of ILC3 and NCR^+^ILC3 cells in DSS‐caused UC mice (Figure 3H). These findings indicated that RFELNs may renew intestinal barrier function by modulating ILC3s.

### 3.4. RFELNs Activated the AhR/IL‐22 Pathway of ILC3s

IL‐22 secretion from ILC3s is predominantly regulated through the aryl hydrocarbon receptor (AhR) activation [41]. Agonist activation of AhR is manifested as nuclear translocation and promotes the level of downstream proteins such as CYP1A1 [42]. As shown in Figure 4A, RFELNs treatment enhanced AhR in the nucleus and its downstream product CYP1A1 protein levels in colon of UC mice.

Figure 4RFELNs activated the AhR/IL‐22 pathway of ILC3s. (A) AhR (cytoplasm and nucleus) and CYP1A1 (cytoplasm) protein expressions were detected using western blot assay in colon. (B) CCK‐8 assay was used to measure the cell viability of MNK3 cells after RFELNs (5 × 103^−5^ × 10^4^ particles/mL) treatment. (C and D) IL‐22 mRNA level was detected by RT‐qPCR and ELISA assays in MNK3 cells after RFELNs (5 × 103^−5^ × 10^4^ particles/mL) treatment. (E) AhR (cytoplasm and nucleus) and CYP1A1 (cytoplasm) protein expressions were detected using western blot assay in MNK3 cells.  ^∗∗^ *p*  < 0.01,  ^∗∗∗^ *p*  < 0.001.

**Figure:**
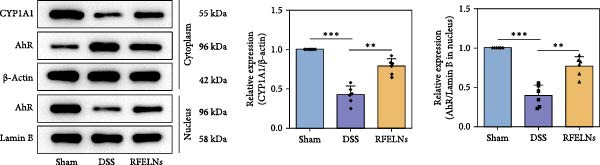
(A)

**Figure:**
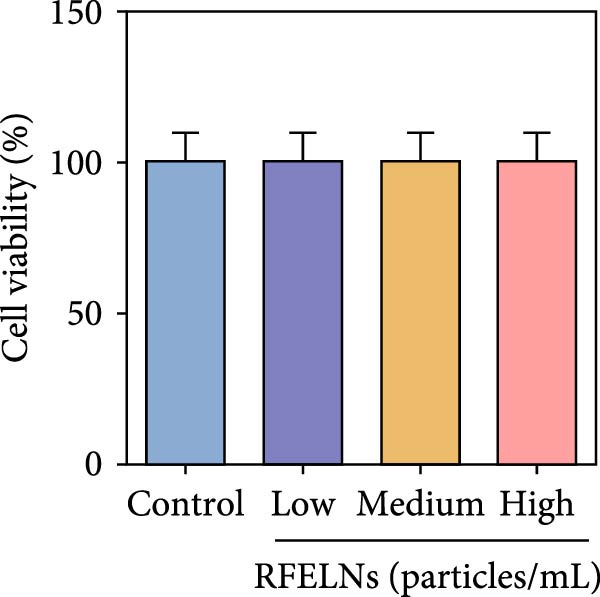
(B)

**Figure:**
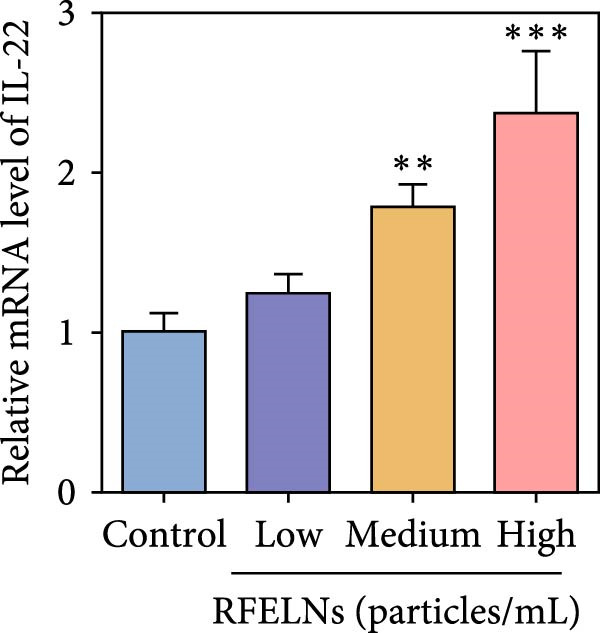
(C)

**Figure:**
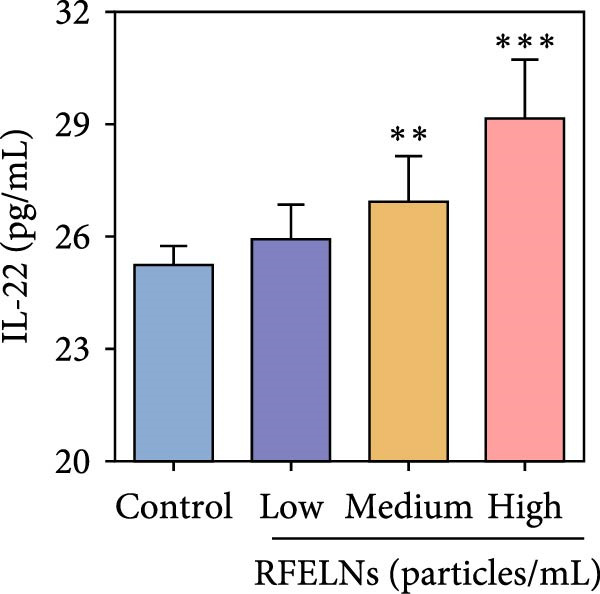
(D)

**Figure:**
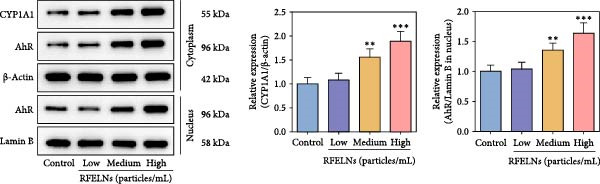
(E)

To further expound whether RFELNs directly modulate ILC3s function via the AHR pathway, we utilized MNK‐3 cells, a mouse lymphoid cell line with ILC3‐specific surface markers and transcription level, which is an ideal in vitro system to study the function of ILC3 [43, 44]. Initially, we conducted CCK‐8 assay on MNK3 cells to ensure the concentration range of RFELNs, which laid the foundation for further research. RFELNs (5 × 10^3^–5 × 10^4^ particles/mL) did not have any toxicity to MNK3 cells (Figure 4B). RT‐qPCR assay detected IL‐22 mRNA levels in RFELNs‐triggered MNK3 cells and observed that RFELNs increased IL‐22 mRNA level (Figure 4C). The results were further verified by ELISA assay in cell culture supernatant (Figure 4D). Next, we observed that RFELNs distinctly increased the level of AhR in the nucleus and CYP1A1 protein levels (Figure 4E).

### 3.5. The Protective Effect of RFELNs on UC Was Dependent on AhR In Vivo

To determine whether RFELNs require activation of the AhR signaling pathway to modulate IL‐22 secretion and thereby confer protection against UC, we inhibited the AhR pathway in UC mice through intraperitoneal administration of CH223191, an AhR antagonist. AhR in the nucleus and CYP1A1 protein levels were enhanced in RFELNs‐treated UC mice, which were counteracted by CH223191 (Figure 5A). CH223191 treatment reversed the beneficial effects of RFELNs on UC symptoms, such as weight gain, increased food intake, increased DAI score, hematoma relief, increased colon length, and improved colon pathology (Figure 5B F). Moreover, CH223191 reversed the influence of RFELNs on the inhibition of TNF‐α, IL‐6, and IL‐1β mRNA levels (Figure 5G). CH223191 disrupted the intestinal barrier recovery induced by RFELNs in DSS‐treated mice, as indicated by changes in FITC‐dextran permeability (Figure 6A), DAO levels (Figure 6B), d‐lactate levels (Figure 6C), Alcian blue staining (Figure 6D), and MUC2 expression and TJ protein levels (Figure 6E). Further, CH223191 suppressed the percentage of ILC3 (Figure 6F) and NCR^+^ILC3 cells (Figure 6G) and RFELNs‐enhanced IL‐22 levels (Figure 6H) and in DSS‐caused UC mice.

Figure 5The protective effect of RFELNs on UC was dependent on AhR in vivo. Mice were randomly assigned to four groups: sham, DSS, RFELNs, and RFELNs+ CH223191. (A) AhR (cytoplasm and nucleus) and CYP1A1 (cytoplasm) protein expressions were detected using western blot assay in different groups, (B) body weight, (C) food intake, (D) DAI score, and (E) colon length were measured in the above groups. (F) HE staining of colon tissues and histological score (Scale bar = 100 μm). (G) TNF‐α, IL‐6, and IL‐1β mRNA levels were measured by RT‐qPCR assay.  ^∗^ *p*  < 0.05,  ^∗∗^ *p*  < 0.01,  ^∗∗∗^ *p*  < 0.001.

**Figure:**
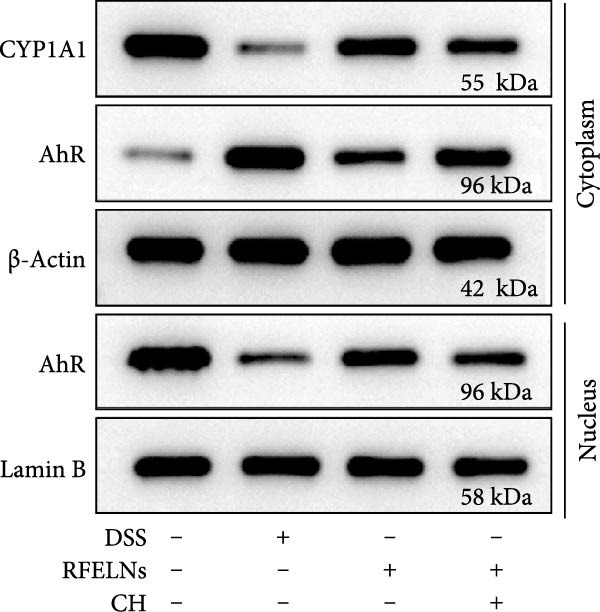
(A)

**Figure:**
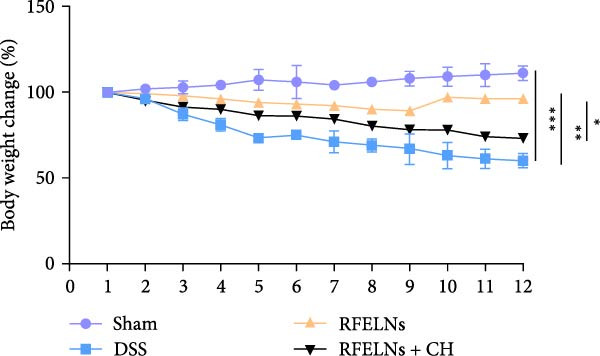
(B)

**Figure:**
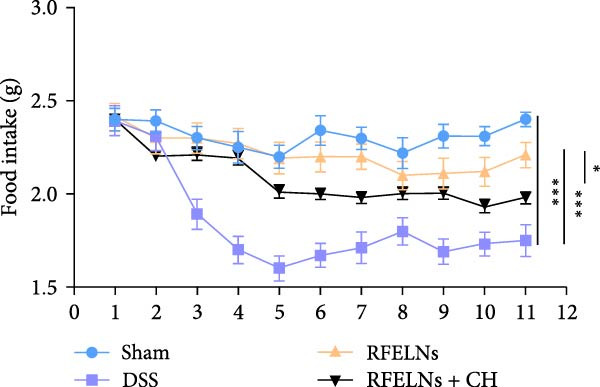
(C)

**Figure:**
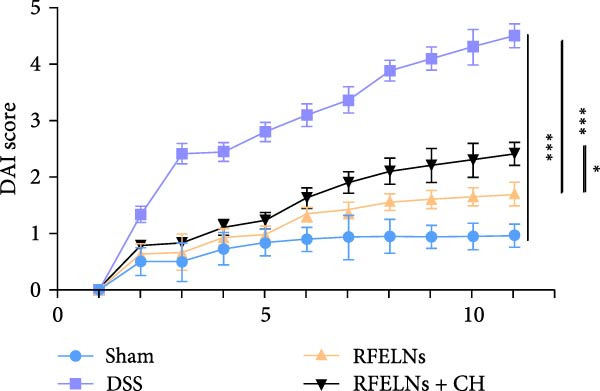
(D)

**Figure:**
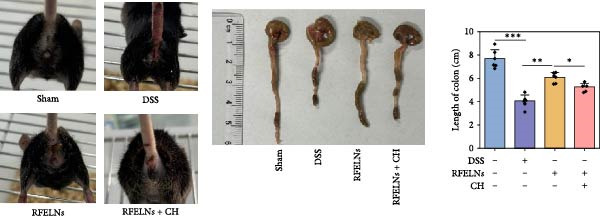
(E)

**Figure:**
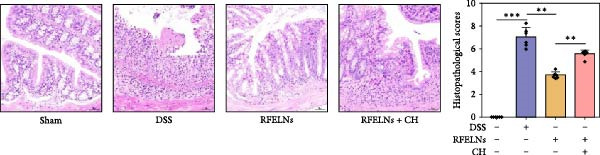
(F)

**Figure:**
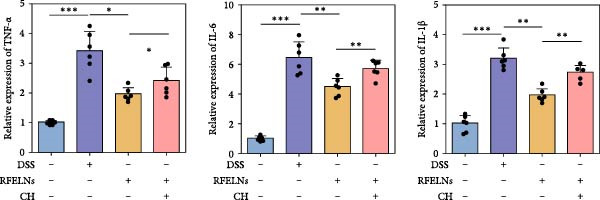
(G)

Figure 6The protective effect of RFELNs on intestinal barrier function in UC mice was dependent on AhR. Mice were randomly assigned to four groups: sham, DSS, RFELNs, and RFELNs+ CH223191. Changes in intestinal permeability were quantified by assessing the ratio of (A) serum FITC‐glucan 4kDA, (B) diamine oxidase (DAO), and (C) D‐LA. (D) Staining of Alcian Blue. (E) MUC2, ZO‐1, and occludin expressions were detected by IHC assays. (F) IL‐22 protein expression was detected by immunofluorescence staining in the colon. (G) Change in the proportion of ILC3 cells in the colon (Scale bar = 100 μm). (H) Change in the proportion of NCR^+^ILC3 cells in the colon.  ^∗^ *p*  < 0.05,  ^∗∗^ *p*  < 0.01,  ^∗∗∗^ *p*  < 0.001.

**Figure:**
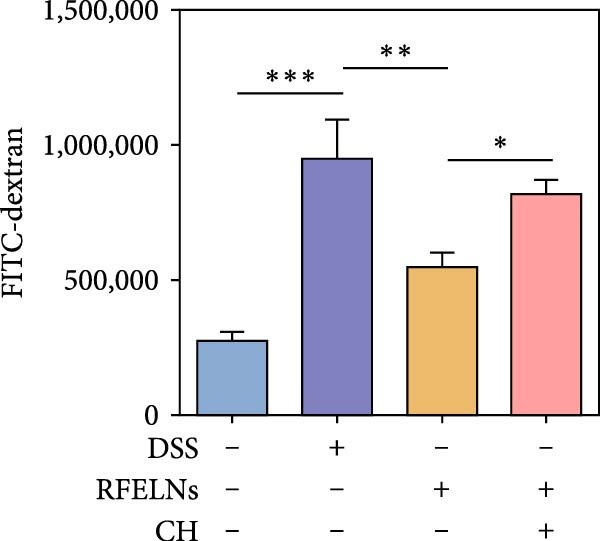
(A)

**Figure:**
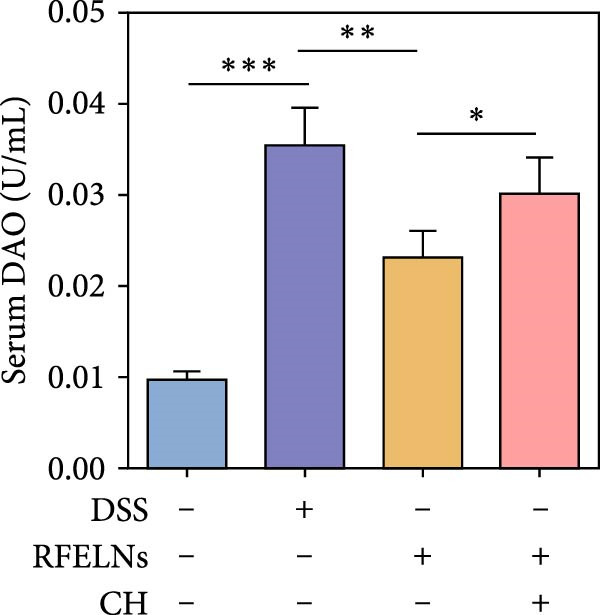
(B)

**Figure:**
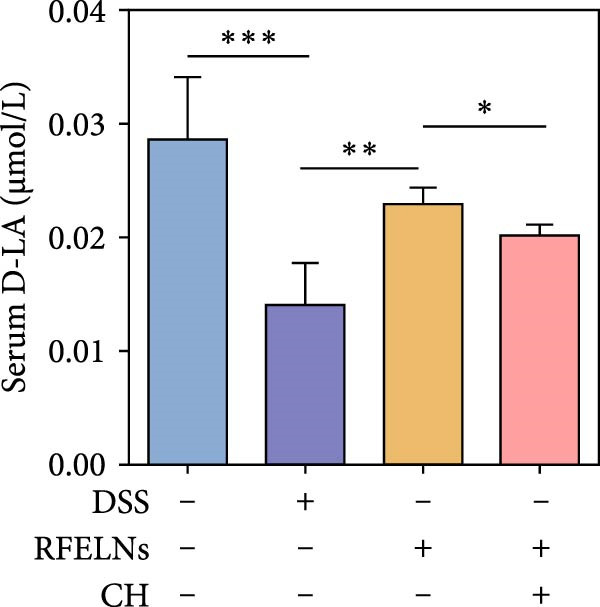
(C)

**Figure:**
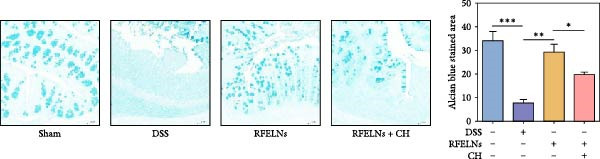
(D)

**Figure:**
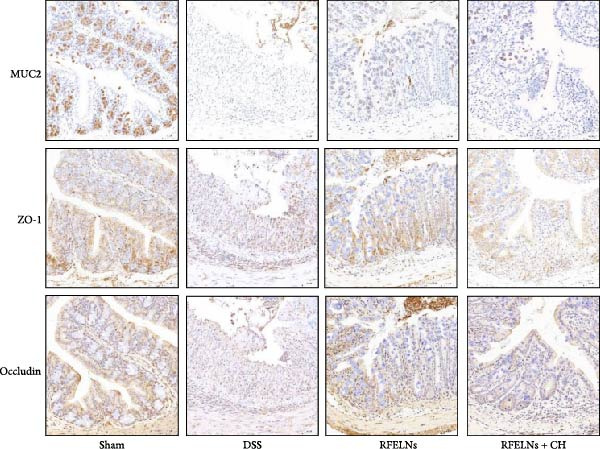
(E)

**Figure:**
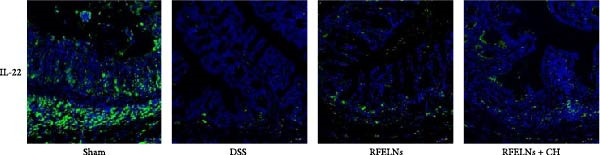
(F)

**Figure:**
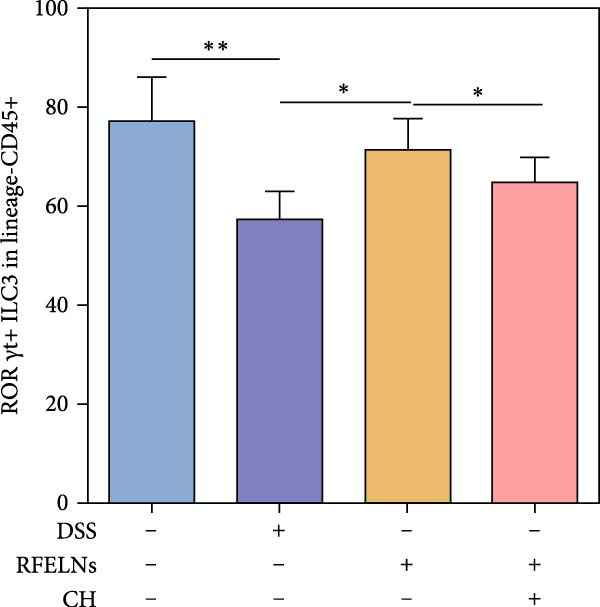
(G)

**Figure:**
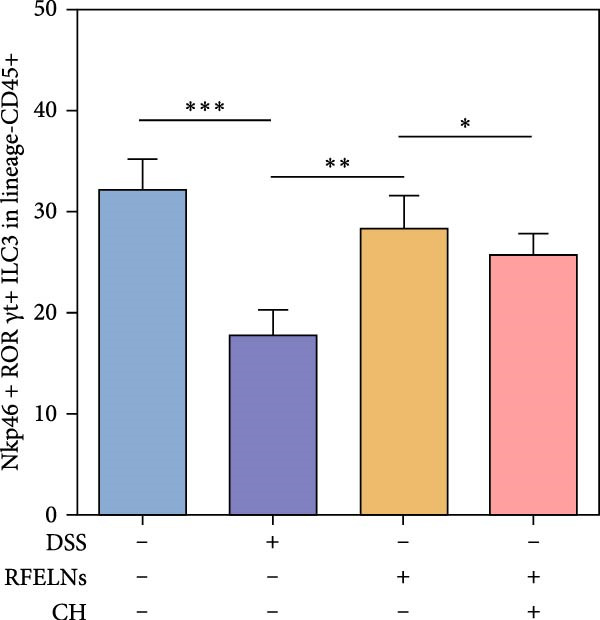
(H)

### 3.6. RFELNs Reduced Inflammation and Enhanced Intestinal Barrier Function Through the AhR Pathway In Vitro

To confirm that RFELNs reduce inflammation and strengthen intestinal barrier function via directly activating the AhR pathway in ILC3, an in vitro culture system using MNK3 and NCM460 cells was developed. We performed CCK‐8 detection on NCM460 cells and found that RFELNs (5 × 10^3^–5 × 10^4^ particles/mL) had no toxic effect on NCM460 cells (Figure 7A). RFELNs at a concentration of 5 × 10^4^ particles/mL were used for further study. As depicted in Figure 7B, MNK3 was co‐cultured with LPS‐stimulated NCM460 cells, resulting in a significant decrease in cell viability. Treatment with RFELNs partially restored cell viability, while the administration of CH223191 completely negated the protective effects of RFELNs on cell viability. Moreover, the levels of IL‐6 and IL‐1β were significantly elevated in the co‐culture of MNK3 and LPS‐treated NCM460 cells. Treatment with RFELNs partially attenuated the inflammatory response, while the addition of CH223191 reversed the anti‐inflammatory effects of RFELNs (Figure 7C). Besides, in the co‐culture of MNK3 and LPS‐treated NCM460 cells, TEER decreased (Figure 7D), dextran permeability increased (Figure 7E), and ZO‐1 and Occludin protein levels were decreased (Figure 7F), all of which were reversed by RFELNs treatment. CH223191 had the opposite effect on intestinal barrier function as RFELNs. Further, CH223191 reversed the effect of RFELNs on IL‐22 (Figure 7G), NCR^+^MFI (Figure 7H), and the level of AhR in the nucleus and CYP1A1 protein levels (Figure 7I). These results suggested that RFELNs inhibit inflammation as well as promote the intestinal barrier by inducing IL‐22 production in MNK‐3 cells in an AhR‐dependent manner.

Figure 7RFELNs reduced inflammation and enhanced intestinal barrier function through the AhR pathway in vitro. (A) CCK‐8 assay was performed to measure NCM460 cell viability after RFELNs (5 × 10^3^–5 × 10^4^ particles/mL) treatment. (B) NCM460 cells were challenged with (10 ng/mL) LPS and cultured with MNK‐3 cells’ supernatant treated with RFELNs alone or together with CH223191 (5 μM), and then cell viability was detected. (C) IL‐6 and IL‐1β levels were measured by ELISA assay in control, LPS, LPS + RFELNs, and LPS + RFELNs + CH groups. (D) Transepithelial electrical resistance (TEER) was detected in control, LPS, LPS + RFELNs, and LPS + RFELNs + CH groups. (E) Epithelial paracellular permeability was measured in the above groups. (F) ZO‐1 and occludin protein levels were measured by western blot assay. (G) Concentrations of IL‐22 in cell culture supernatant were evaluated by ELISA. (H) Flow cytometry assay to evaluate mean fluorescence intensity (MFI) of NCR in RORγt^+^MNK3 cells. (I) AhR (cytoplasm and nucleus) and CYP1A1 (cytoplasm) protein expressions were detected using western blot assay in different groups.  ^∗^ *p*  < 0.05,  ^∗∗^ *p*  < 0.01,  ^∗∗∗^ *p*  < 0.001.

**Figure:**
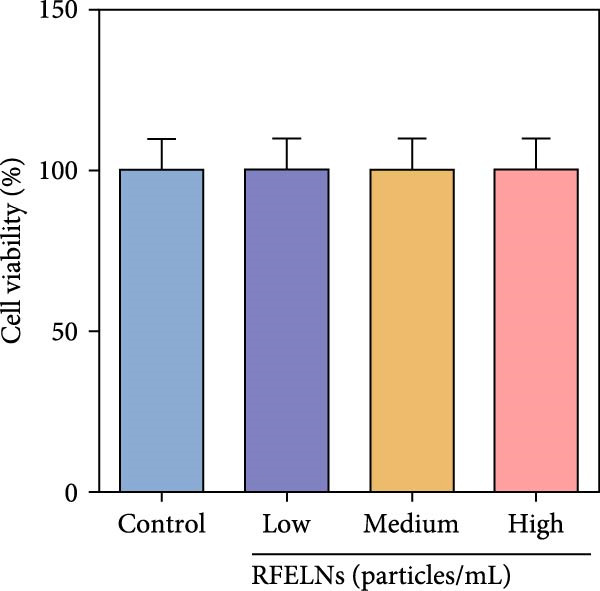
(A)

**Figure:**
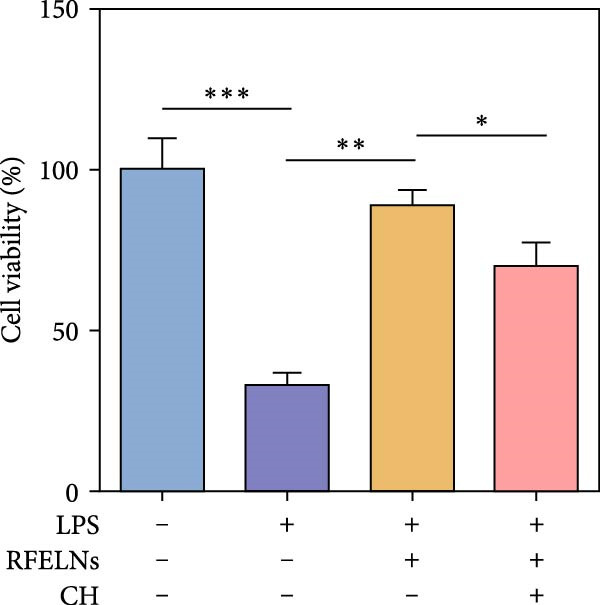
(B)

**Figure:**
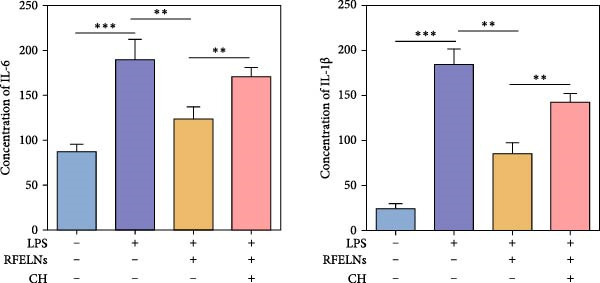
(C)

**Figure:**
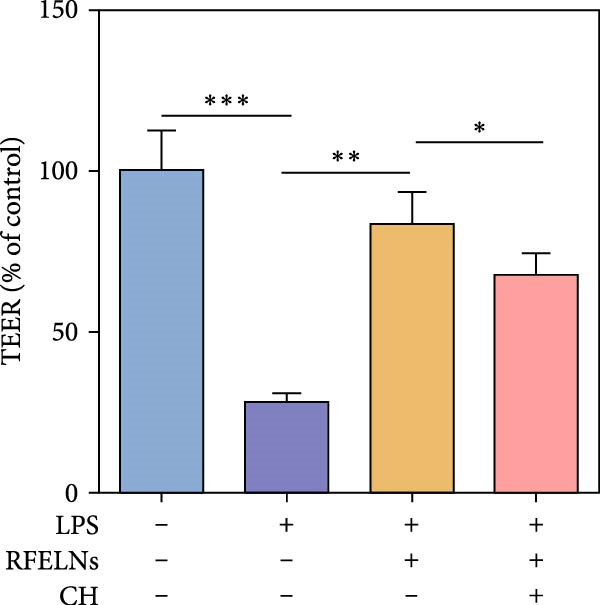
(D)

**Figure:**
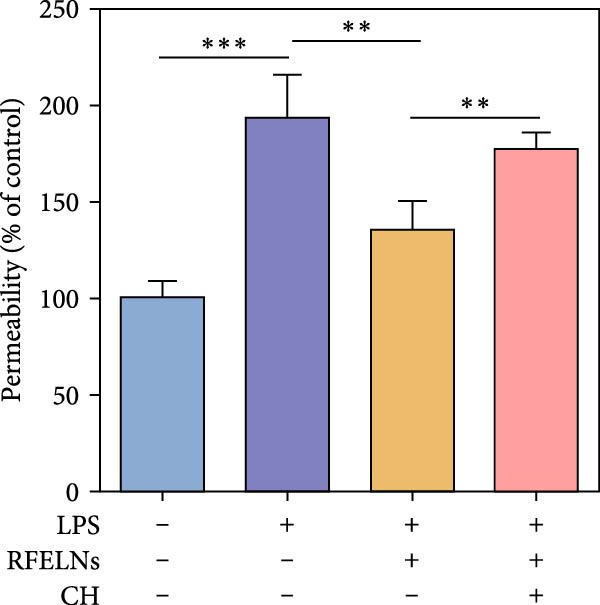
(E)

**Figure:**
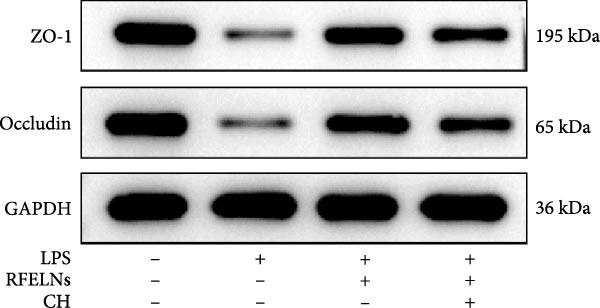
(F)

**Figure:**
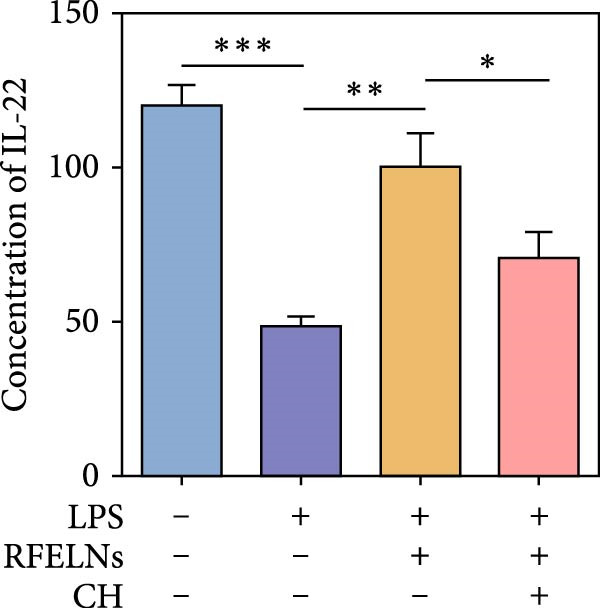
(G)

**Figure:**
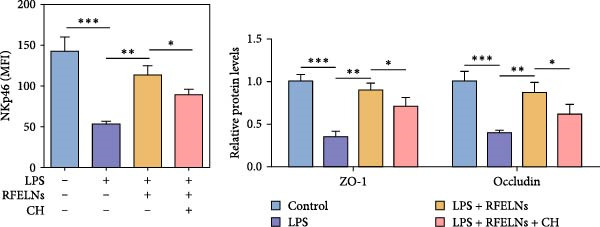
(H)

**Figure:**
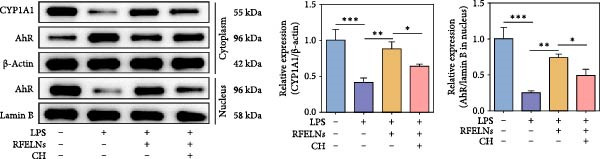
(I)

### 3.7. Modulating Gut Microbiota by RFELNs

Gut microbiota plays a vital role in modulating host metabolism, immunity, and intestinal barrier function [45]. We carried out 16S rRNA gene sequencing to examine the gut microbiota composition in mice from three different groups. As depicted in Figure 8A, RFELNs significantly restored the Chao1, Shannon, and Simpson indices of gut microbiota in UC mice, with notable statistical differences compared to the model group, implying that RFELNs regulate α‐diversity of gut microbiota. PCoA analysis showed that there was a significant separation between the DSS group and the sham group, while there was overlap between the RFELNs group and both groups, suggesting that RFELNs regulated the gut microbiota composition of UC mice and restored it to normal (Figure 8B). Further analysis of species abundance at the phylum level showed distinct differences between the DSS and RFELNs groups. The abundance of Bacteroidetes (phylum) and Firmicutes (phylum), was reduced, and Proteobacteria (phylum) was increased after induction by DSS, and administration of RFELNs abolished DSS‐caused effects on these species (Figure 8C). To better understand the differences in microbial composition, LEfSe was conducted. Using LDA > 4 as the cut‐off value, the results showed that the contribution of Firmicutes was greater in the control group, Proteobacteria had a greater impact in the UC group, and Tannerellaceae had a greater influence in RFELNs group (Figures 8D,E). In summary, the therapeutic effect of RFELNs on UC is partly due to its modulation of the gut microbiota.

Figure 8Modulating gut microbiota by RFELNs. (A) Changes in the (A) Changes in the Chao1, Shannon, and Simpson index of gut microbiota. (B) PCoA analysis of three groups of gut microbiota. (C) Bar plot of community at the phylum level. (D, E) Linear discriminant analysis effect size (LEfSe) and cladogram based on LEfSe analysis.  ^∗^ *p*  < 0.05,  ^∗∗^ *p*  < 0.01.

**Figure:**
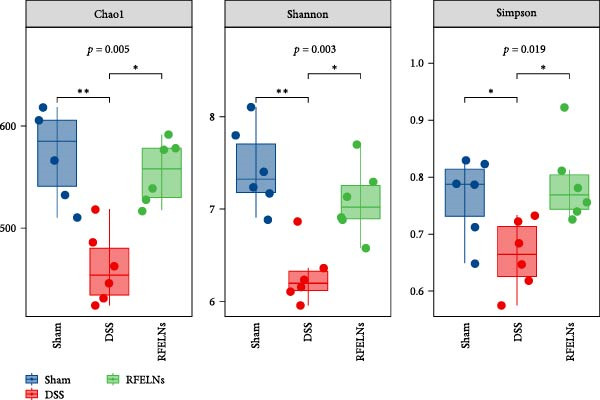
(A)

**Figure:**
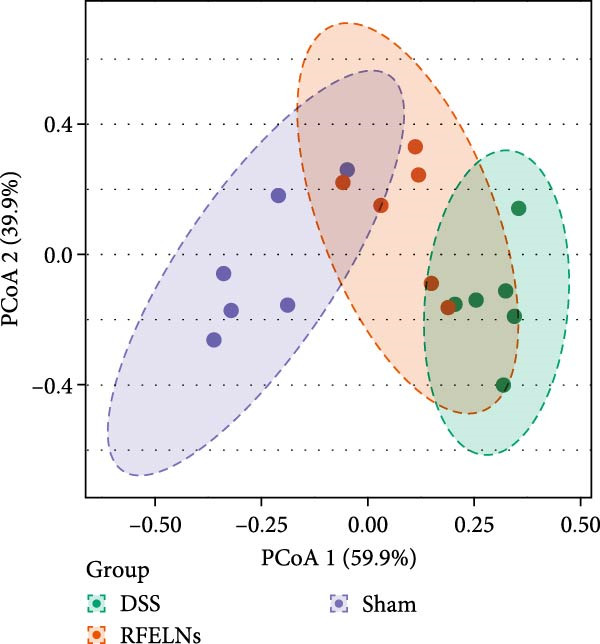
(B)

**Figure:**
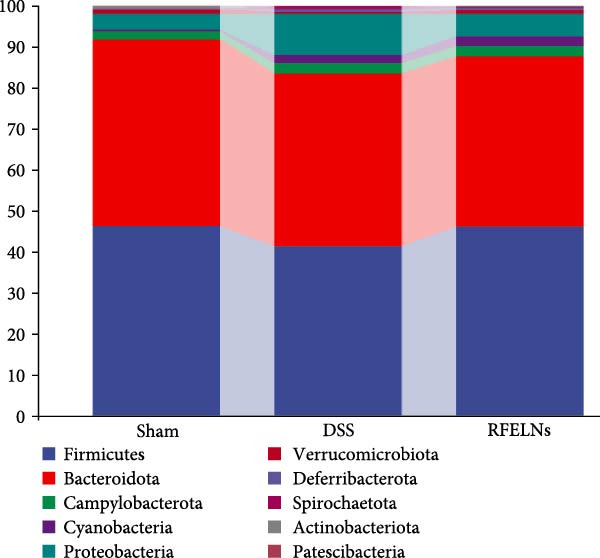
(C)

**Figure:**
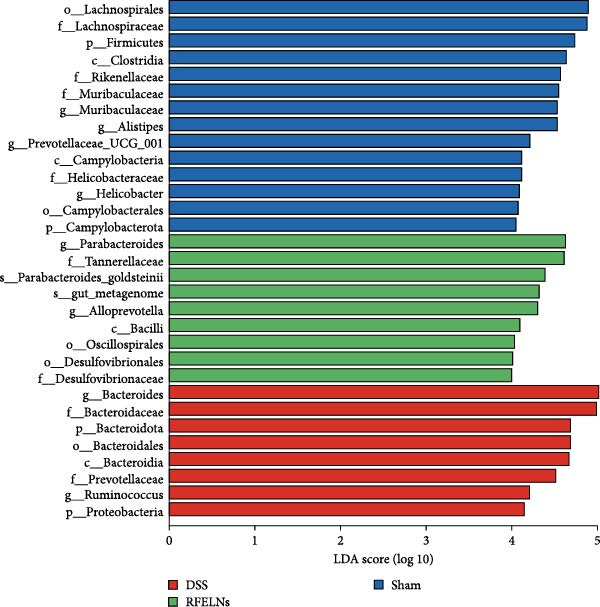
(D)

**Figure:**
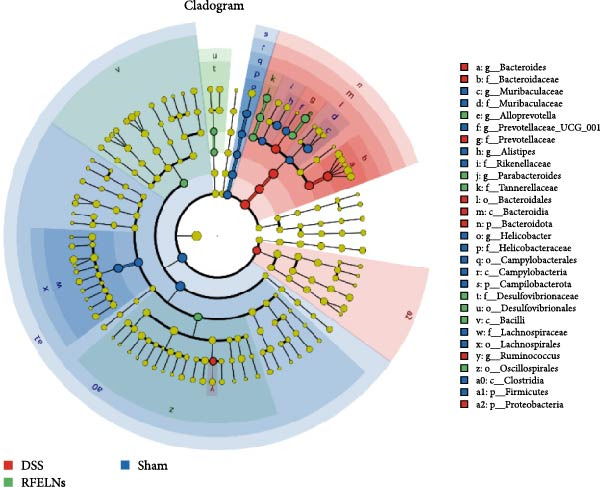
(E)

### 3.8. RFELNs Modulated Gut Microbiota‐Derived Tryptophan Metabolism

To further probe the effects of RFELNs on intestinal microbial metabolites after regulating intestinal microbiota composition, we utilized fecal metabolomics analyses. The PCA results revealed significant changes in the metabolite composition of the DSS group, indicating that gut microbiota metabolites were changed by DSS induction. The RFELNs treatment group overlapped with the DSS group (Figure 9A). Moreover, the levels of indoles and their derivatives varied differently between the DSS and RFELNs groups (Figure 9B). Notably, The L‐tryptophan level in the DSS group was declined and the concentrations of indole derivatives, including IA, IAA, IPA, and IAld exhibited a reduction (Figure 9C–H). The data revealed a disruption in tryptophan metabolism linked to the gut microbiota in UC mice. However, RFELNs treatment, compared to the DSS group, restored the levels of these metabolites, suggesting that RFELNs may have therapeutic potential by modulating gut microbiota‐associated tryptophan metabolism. According to previous research, these tryptophan metabolites act as specific ligands for AhR and serve as major activators of the AhR pathway [46, 47]. The results suggested that RFELNs may promote the production of endogenous ligands of intestinal AhR by modulating tryptophan metabolism, thus activating AhR to inhibit inflammation and restore the intestinal barrier, thus slowing down the progression of UC.

Figure 9Regulation of gut microbial metabolism by RFELNs. (A) The principal component analysis of the control group, DSS group, and RFELNs groups. (B) Volcano plot of DSS compared with RFELNs. (C) Metabolite changes were compared between groups. (D–H) Quantification of tryptophan and its metabolites in mice feces. IA, indole acrylic acid; IAA, Indole‐3‐acetic acid; IAld, Indole‐3‐carboxaldehyde; IPA, 3‐Indolepropionic acid. ^∗∗^ *p*  < 0.01,  ^∗∗∗^ *p*  < 0.001.

**Figure:**
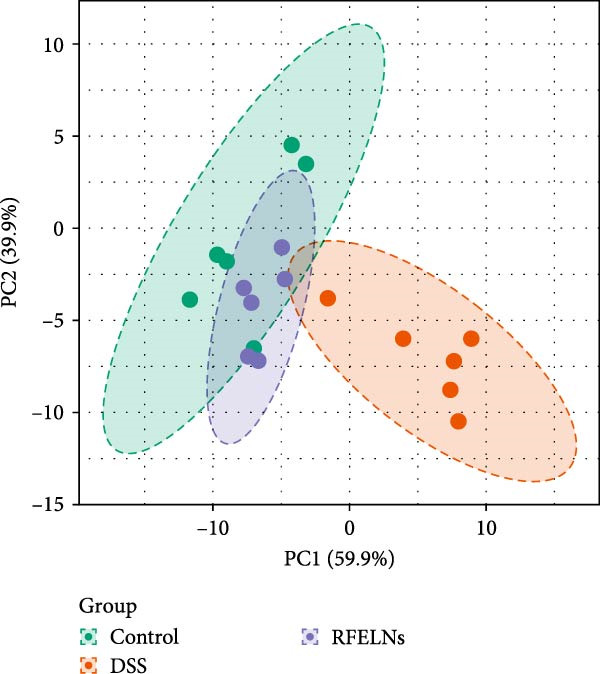
(A)

**Figure:**
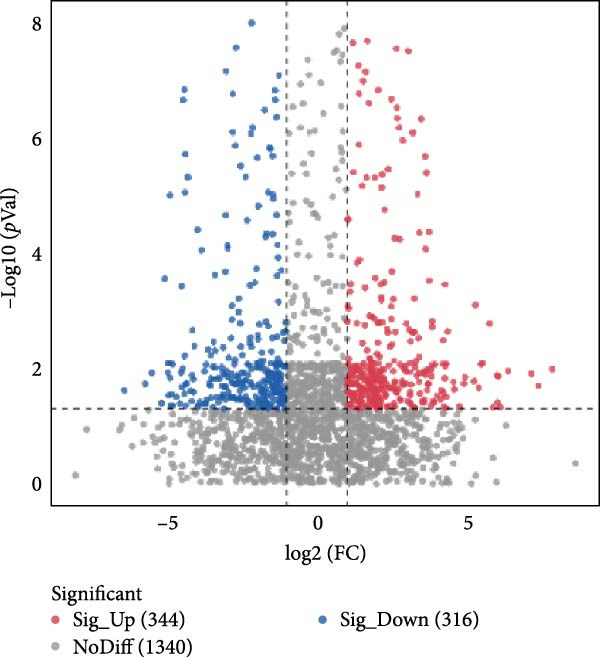
(B)

**Figure:**
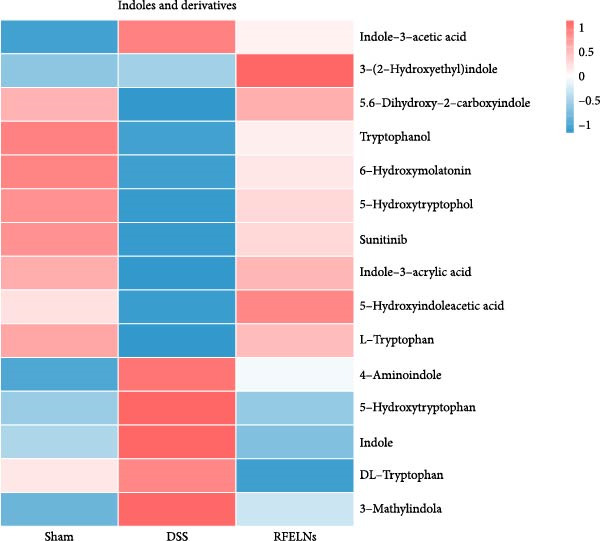
(C)

**Figure:**
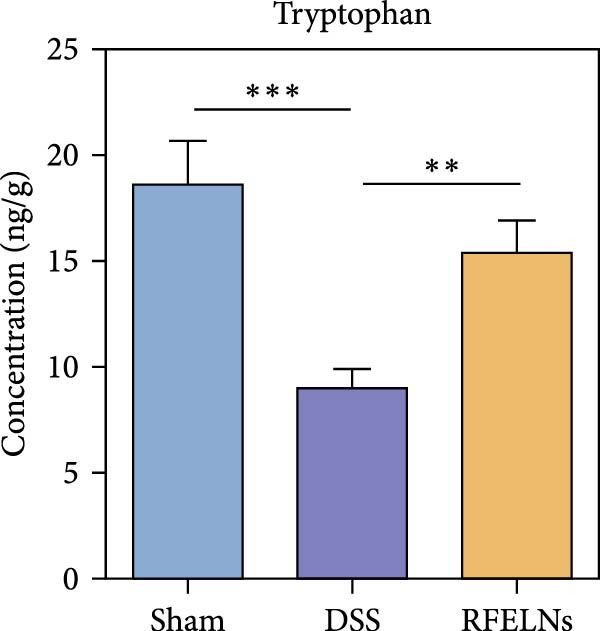
(D)

**Figure:**
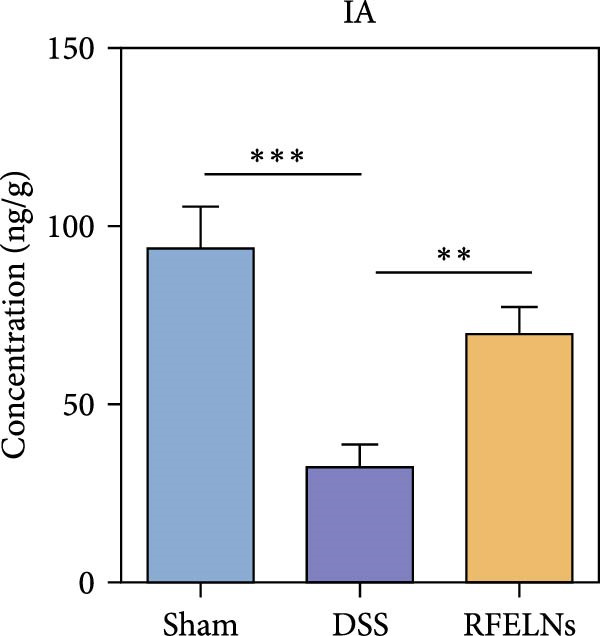
(E)

**Figure:**
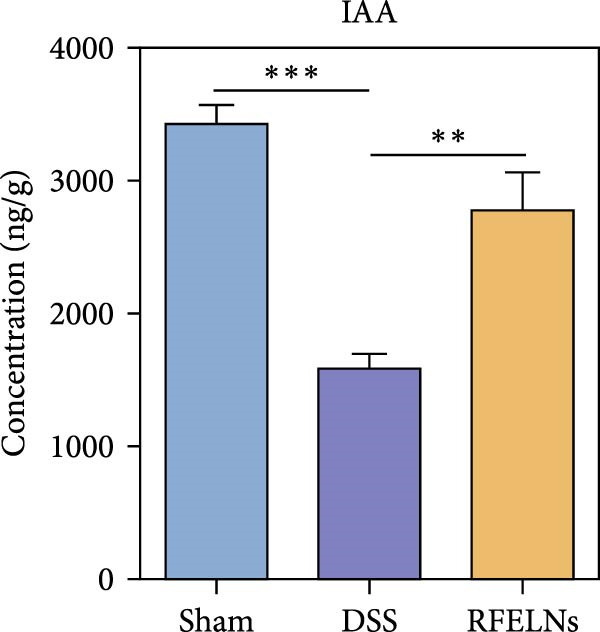
(F)

**Figure:**
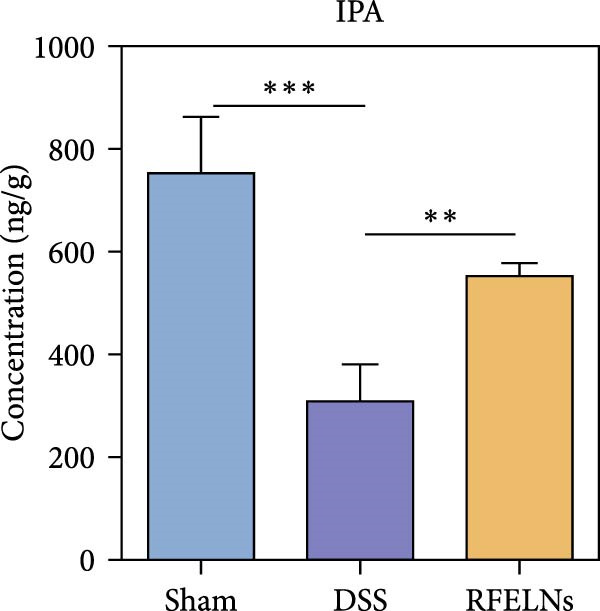
(G)

**Figure:**
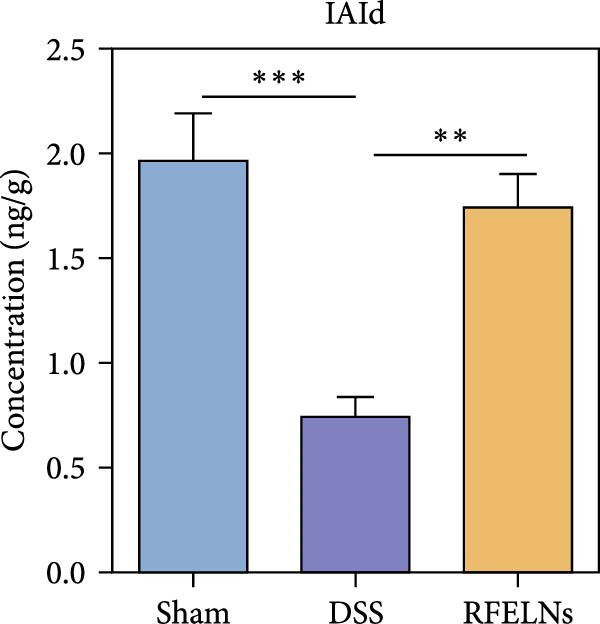
(H)

## 4. Discussion

In recent years, PELNs have attracted considerable attention due to their excellent biocompatibility, low toxicity, and potent biological activity [30]. Due to its high yield and purity, PELNs have been widely studied in a variety of diseases, including inflammatory bowel diseases such as colitis [28, 30]. In this work, for the first time, we explored the effects of RFELNs extracted from *Robinia pseudoacacia* L. flowers on UC. RFELNs activated AhR by regulating tryptophan metabolites associated with intestinal flora. Mechanically, RFELNs can activate AHR signaling and promote ILC3s secretion of IL‐22, thereby enhancing intestinal epithelial TJ expression and mucus secretion and effectively repairing intestinal mucosal injury.

At the beginning of this study, we successfully isolated RFELNs and evaluated their pharmacological effects. The results indicated that RFELNs significantly reversed DSS‐induced pathological changes in the colon. Dysfunction of the intestinal epithelial barrier is increasingly recognized as a critical contributor to the pathogenesis of UC [48]. Therefore, repairing the damaged intestinal mucosa and promoting mucosal healing have become central goals in UC treatment. The intestinal barrier consists of the mucus layer, epithelial cells, and TJ proteins, which together regulate nutrient absorption and prevent the invasion of toxins and pathogens [49]. The mucus layer is composed of mucin (MUC2) released by epithelial goblet cells and is the first line of defense against pathogen invasion. Xu et al. [32] reported that chronic DSS‐induced colitis is associated with reduced MUC2 levels and a decrease in goblet cell numbers. TJs are a multi‐protein complex that regulates paracellular permeability in the intestinal epithelium, serving as an indicator of gut health and epithelial barrier integrity [50]. Previous studies have confirmed that TJ proteins, including ZO‐1 and Occludin, are significantly down‐regulated in UC mice [51]. Consistent with these findings, we observed that DSS disrupted the intestinal barrier, whereas RFELNs treatment effectively restored its integrity.

IL‐22 has received considerable attention for its role in promoting mucosal healing and repairing tissue damage [52]. IL‐22 has received considerable attention for its role in promoting mucosal healing and repairing tissue damage [53, 54]. Studies have shown that IL‐22‐deficient mice exhibit more severe intestinal barrier dysfunction and delayed wound healing upon DSS induction compared to wild‐type mice [55]. ILC3s, primarily located in the intestinal lamina propria, are the main producers of IL‐22 [9]. We hypothesized that the protective effects of RFELNs on the intestinal epithelial barrier may be mediated through the ILC3–IL‐22 axis. Our results showed that RFELNs recover IL‐22 levels and increased the proportions of ILC3 and NCR^+^ILC3 cells in UC mice. Consistent with in vivo findings, RFELNs also upregulated IL‐22 expression in MNK‐3 cells in vitro. However, the mechanism by which RFELNs enhance IL‐22 production by ILC3s remains unclear. Li et al. [56] indicated that Baicalein enhances IL‐22 production by directly activating AhR in ILC3s. Similarly, administration of FICZ, an AhR agonist, promoted IL‐22 secretion and significantly ameliorated chemically induced colitis, while AhR antagonists worsened disease severity [18]. Moreover, Zhang et al. [57] demonstrated that activation of the AHR/IL‐22 signaling improves intestinal barrier function and significantly alleviates DSS‐induced colitis. Based on these findings, we hypothesized that the protective effects of RFELNs against UC are mediated through the AhR pathway. The nuclear translocation of AhR and increased expression of its downstream target gene, CYP1A1, provide direct evidence that RFELNs activate the AhR pathway in UC models. Both in vivo and in vitro studies confirmed that RFELNs stimulate IL‐22 production by ILC3s via an AhR‐dependent mechanism, thereby suppressing inflammation and enhancing intestinal barrier integrity.

The gut microbiota plays a crucial role in the pathogenesis of UC by influencing energy metabolism, regulating immune homeostasis, and maintaining intestinal barrier integrity [58]. Structural alterations in the intestinal bacterial community can lead to microbial dysbiosis, thereby exacerbating the inflammatory response [59]. Both UC patients and animal models exhibit similar microbial changes, characterized by reduced abundance and diversity, as well as an imbalance between beneficial and pathogenic microorganisms [18]. Consistent with these findings, our study demonstrated that mice with colitis showed a significant decline in gut microbial diversity, accompanied by a reduction in beneficial bacteria (e.g., Bacteroidetes) and an increase in harmful bacteria (e.g., Proteobacteria). RFELNs treatment effectively ameliorated gut dysbiosis in UC mice. These results suggested that RFELNs may help restore intestinal homeostasis by reshaping the gut microbiota structure.

Beyond their direct impact, many microbial products also exert indirect influence host gut immunity [60]. Among these, tryptophan metabolites produced by gut microbiota play a crucial role in maintaining intestinal homeostasis [61]. The gut microbiota converts tryptophan, an essential amino acid, into indole and its derivatives through various metabolic pathways [62]. Indole‐3‐acetic acid (IAA) is produced by a wide range of bacterial genera, including Lactobacillus, Clostridium, Bacteroides, and Bifidobacterium [63]. Lactobacillus species can convert tryptophan into indole‐3‐aldehyde (IAld) via indolelactate dehydrogenase and aromatic amino acid transaminase. Certain species within the Bacteroides and Clostridium genera are capable of producing indole‐3‐propionic acid (IPA) and indoleacrylic acid (IA) [64]. Importantly, our experimental results demonstrated that RFELNs treatment increased the abundance of Bacteroidetes, Mucispirillum, and Lactobacillus, all of which are involved in bacterial tryptophan metabolism. As expected, RFELNs intervention significantly elevated the levels of L‐tryptophan and its metabolites—including IA, IAA, IPA, and IAld—in UC mice. Several of these indole derivatives (IA, IAA, IPA, and IAld) have been identified as endogenous ligands and agonists of the AhR [65, 66]. These findings demonstrated that RFELNs alleviate UC by activating the AhR pathway via gut microbiota‐mediated tryptophan metabolism.

**Figure 10:**
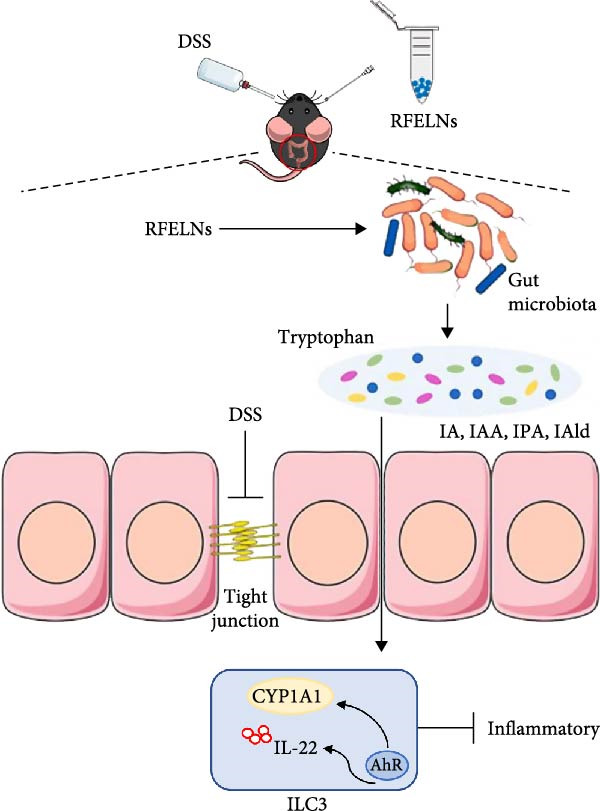
Schematic diagram illustrating the proposed mechanism by which RFELNs alleviate ulcerative colitis via modulation of gut microbiota‐related tryptophan metabolism and activation of the AhR/IL‐22 signaling axis.

In conclusion, our findings demonstrated that RFELNs attenuate inflammation and restore intestinal barrier function in UC models via an AhR‐dependent pathway. Furthermore, we confirmed that RFELNs alleviate UC by modulating the gut microbiota composition and enhancing the production of key microbial tryptophan metabolites—IA, IAA, IPA, and IAld—which activate the AhR signaling pathway and subsequently promote IL‐22 secretion by ILC3s (Figure 10). This study provides new insights into potential therapeutic strategies for UC.

## Ethics Statement

This study was approved by The First People’s Hospital of Changzhou.

## Disclosure

All authors read and approved the final manuscript. The authors take full responsibility for the content of the publication.

## Conflicts of Interest

The authors declare no conflicts of interests.

## Author Contributions

Feihan Shen and Kewen Sun designed this study. Jianguo Song and Xueping Chen performed all the experiments and analyzed the data and prepared the figures. Juan Dai and Liwen Zhang drafted the initial manuscript. Ying Qi and Liang Ma reviewed and revised the manuscript. Feihan Shen, Kwen Sun and Jianguo Song made equal contributions to this study.

## Funding

This study was sponsored by grants from the Top Talent of Changzhou “The 14th Five‐Year Plan” High‐Level Health Talents Training Project (2022CZBJ006; 2024BJHB013), The National Natural Science Foundation of China (No. 81700500; 82400025), Youth Science and Technology Project of Changzhou Municipal Health Commission (QN202349; QN202348; QN202316), the Research Project of Changzhou Medical Center, Nanjing Medical University (CMCB202301; CMCB202423), Natural Science Foundation of Xinjiang Uygur Autonomous Region (2022D01A307), Xinjiang Kirgiz Autonomous Prefecture Medical and Health Technology Project (Grant No. 43; No. 44), and Changzhou Applied Basic Research Program (CJ20220091).

## Data Availability

The data sets used and/or analyzed during the current study are available from the corresponding author upon reasonable request.

## References

[1] Høivik M. L. , Moum B. , Solberg I. C. , Henriksen M. , Cvancarova M. , and Bernklev T. , Work Disability in Inflammatory Bowel Disease Patients 10 Years After Disease Onset: Results From the IBSEN Study, Gut. (2013) 62, no. 3, 368–375, 10.1136/gutjnl-2012-302311, 2-s2.0-84873407754. 22717453

[2] Burisch J. , Zhao M. , and Odes S. , et al.The Cost of Inflammatory Bowel Disease in High-Income Settings, Lancet Gastroenterology & Hepatology Commission. (2023) 8, no. 5, 458–492. 10.1016/S2468-1253(23)00003-1 36871566

[3] Ng S. C. , Shi H. Y. , and Hamidi N. , et al.Worldwide Incidence and Prevalence of Inflammatory Bowel Disease in the 21st Century: A Systematic Review of Population-Based Studies, The Lancet. (2017) 390, no. 10114, 2769–2778, 10.1016/S0140-6736(17)32448-0, 2-s2.0-85031499214. 29050646

[4] Zhou R. , Huang K. , and Chen S. , et al.Zhilining Formula Alleviates DSS-Induced Colitis Through Suppressing Inflammation and Gut Barrier Dysfunction via the AHR/NF-κBp65 Axis, Phytomedicine. (2024) 129, 10.1016/j.phymed.2024.155571, 155571. 38677270

[5] Feuerstein J. D. and Cheifetz A. S. , Ulcerative Colitis, Mayo Clinic Proceedings. (2014) 89, no. 11, 1553–1563, 10.1016/j.mayocp.2014.07.002, 2-s2.0-84914104629. 25199861

[6] Magro F. , Gionchetti P. , and Eliakim R. , et al.Third European Evidence-Based Consensus on Diagnosis and Management of Ulcerative Colitis. Part 1: Definitions, Diagnosis, Extra-Intestinal Manifestations, Pregnancy, Cancer Surveillance, Surgery, and Ileo-Anal Pouch Disorders, Journal of Crohn’s and Colitis. (2017) 11, no. 6, 649–670, 10.1093/ecco-jcc/jjx008, 2-s2.0-85015740067. 28158501

[7] Shaw K. A. , M. Bertha , and T. Hofmekler , et al.Dysbiosis, Inflammation, and Response to Treatment: A Longitudinal Study of Pediatric Subjects With Newly Diagnosed Inflammatory Bowel Disease, Genome Medicine. (2016) 8, no. 1, 10.1186/s13073-016-0331-y, 2-s2.0-84979498467, 75. 27412252 PMC4944441

[8] Domingues R. G. and Hepworth M. R. , Immunoregulatory Sensory Circuits in Group 3 Innate Lymphoid Cell (ILC3) Function and Tissue Homeostasis, Frontiers in Immunology. (2020) 11, 10.3389/fimmu.2020.00116, 116. 32117267 PMC7015949

[9] Zhou J. , Yue J. , and Yao Y. , et al.Dihydromyricetin Protects Intestinal Barrier Integrity by Promoting IL-22 Expression in ILC3s Through the AMPK/SIRT3/STAT3 Signaling Pathway, Nutrients. (2023) 15, no. 2, 10.3390/nu15020355, 355. 36678226 PMC9861697

[10] De Juan A. and Segura E. , Modulation of Immune Responses by Nutritional Ligands of Aryl Hydrocarbon Receptor, Frontiers in Immunology. (2021) 12, 10.3389/fimmu.2021.645168. PMC8173196 34093534

[11] Zeng B. , Shi S. , Ashworth G. , Dong C. , Liu J. , and Xing F. , ILC3 Function as a Double-Edged Sword in Inflammatory Bowel Diseases, Cell Death & Disease. (2019) 10, no. 4, 10.1038/s41419-019-1540-2, 2-s2.0-85064078658, 315. 30962426 PMC6453898

[12] Gronke K. , Hernandez P. P. , and Zimmermann J. , et al.Interleukin-22 Protects Intestinal Stem Cells Against Genotoxic Stress, Nature. (2019) 566, no. 7743, 249–253, 10.1038/s41586-019-0899-7, 2-s2.0-85061504994. 30700914 PMC6420091

[13] Li S. , Heller J. J. , and Bostick J. W. , et al.Ikaros Inhibits Group 3 Innate Lymphoid Cell Development and Function by Suppressing the Aryl Hydrocarbon Receptor Pathway, Immunity. (2016) 45, no. 1, 185–197, 10.1016/j.immuni.2016.06.027, 2-s2.0-84990869187. 27438771 PMC4959810

[14] Xiao W. , Son J. , Vorrink S. U. , Domann F. E. , and Goswami P. C. , Ligand-Independent Activation of Aryl Hydrocarbon Receptor Signaling in PCB3-Quinone Treated HaCaT Human Keratinocytes, Toxicology Letters. (2015) 233, no. 3, 258–266, 10.1016/j.toxlet.2015.02.005, 2-s2.0-84922608563. 25668756 PMC4341842

[15] Maier A. M. , Huth K. , and Alessandrini F. , et al.The Aryl Hydrocarbon Receptor Regulates Lipid Mediator Production in Alveolar Macrophages, Frontiers in Immunology. (2023) 14, 10.3389/fimmu.2023.1157373, 1157373. 37081886 PMC10110899

[16] Wang X. , Huang S. , and Zhang M. , et al.Gegen Qinlian Decoction Activates AhR/IL-22 to Repair Intestinal Barrier by Modulating Gut Microbiota-Related Tryptophan Metabolism in Ulcerative Colitis Mice, Journal of Ethnopharmacology. (2023) 302, no. Pt B, 10.1016/j.jep.2022.115919, 115919. 36356716

[17] Deng Y. , Hou X. , Wang H. , Du H. , and Liu Y. , Influence of Gut Microbiota-Mediated Immune Regulation on Response to Chemotherapy, Pharmaceuticals. (2024) 17, no. 5, 10.3390/ph17050604, 604. 38794174 PMC11123941

[18] Huang S. , Ye Q. , Wang A. , and Chen Y. , Paeoniae Decoction Restores Intestinal Barrier Dysfunction by Promoting the Interaction Between ILC3 and Gut Flora, Phytomedicine. (2024) 132, 10.1016/j.phymed.2024.155873, 155873. 39024673

[19] Lee S. H. , Lee J. A. , Shin M. R. , Park H. J. , and Roh S. S. , Citrus unshiu Peel Attenuates Dextran Sulfate Sodium-Induced Ulcerative Colitis in Mice due to Modulation of the PI3K/Akt Signaling Pathway and MAPK and NF-κB, Evidence-Based Complementary and Alternative Medicine. (2022) 2022, no. 13, 10.1155/2022/4041402, 4041402. 35620406 PMC9129974

[20] He X. Q. , Liu D. , and Liu H. Y. , et al.Prevention of Ulcerative Colitis in Mice by Sweet Tea (*Lithocarpus litseifolius*) via the Regulation of Gut Microbiota and Butyric-Acid-Mediated Anti-Inflammatory Signaling, Nutrients. (2022) 14, no. 11, 10.3390/nu14112208, 2208. 35684007 PMC9183097

[21] Yue B. , Ren J. , and Yu Z. , et al.Pinocembrin Alleviates Ulcerative Colitis in Mice via Regulating Gut Microbiota, Suppressing Tlr4/md2/nf-κb Pathway and Promoting Intestinal Barrier, Bioscience Reports. (2020) 40, no. 7, 10.1042/BSR20200986. PMC7391130 32687156

[22] Wong C. B. , Tanaka A. , Kuhara T. , and Xiao J.-Z. , Potential Effects of Indole-3-Lactic Acid, a Metabolite of Human Bifidobacteria, on NGF-Induced Neurite Outgrowth in PC12 Cells, Microorganisms. (2020) 8, no. 3, 10.3390/microorganisms8030398, 398. 32178456 PMC7143819

[23] Jo Y. J. , Tagele S. B. , and Pham H. Q. , et al.In Situ Profiling of the Three Dominant Phyla Within the Human Gut Using TaqMan PCR for Pre-Hospital Diagnosis of Gut Dysbiosis, International Journal of Molecular Sciences. (2020) 21, no. 6, 10.3390/ijms21061916, 1916. 32168885 PMC7139488

[24] Motti R. , Paura B. , Cozzolino A. , and Falco B. , Edible Flowers Used in Some Countries of the Mediterranean Basin: An Ethnobotanical Overview, Plants. (2022) 11, no. 23, 10.3390/plants11233272, 3272. 36501312 PMC9736219

[25] Kim J. , Li S. , Zhang S. , and Wang J. , Plant-Derived Exosome-Like Nanoparticles and Their Therapeutic Activities, Asian Journal of Pharmaceutical Sciences. (2022) 17, no. 1, 53–69, 10.1016/j.ajps.2021.05.006. 35261644 PMC8888139

[26] Di Gioia S. , Hossain M. N. , and Conese M. , Biological Properties and Therapeutic Effects of Plant-Derived Nanovesicles, Open Medicine. (2020) 15, no. 1, 1096–1122, 10.1515/med-2020-0160. 33336066 PMC7718644

[27] Deng Z. , Rong Y. , and Teng Y. , et al.Broccoli-Derived Nanoparticle Inhibits Mouse Colitis by Activating Dendritic Cell AMP-Activated Protein Kinase, Molecular Therapy. (2017) 25, no. 7, 1641–1654, 10.1016/j.ymthe.2017.01.025, 2-s2.0-85021844814. 28274798 PMC5498816

[28] Zhu M. Z. , Xu H. M. , and Liang Y. J. , et al.Edible Exosome-Like Nanoparticles from *portulaca oleracea* L Mitigate DSS-Induced Colitis via Facilitating Double-Positive CD4(+)CD8(+)T Cells Expansion, Journal of Nanobiotechnology. (2023) 21, no. 1, 10.1186/s12951-023-02065-0, 309. 37653406 PMC10469825

[29] Sriwastva M. K. , Deng Z. B. , and Wang B. , et al.Exosome-Like Nanoparticles From Mulberry Bark Prevent DSS-Induced Colitis via the AhR/COPS8 Pathway, EMBO reports. (2022) 23, no. 3, 10.15252/embr.202153365. PMC8892346 34994476

[30] Kim J. , Zhang S. , Zhu Y. , Wang R. , and Wang J. , Amelioration of Colitis Progression by Ginseng-Derived Exosome-Like Nanoparticles Through Suppression of Inflammatory Cytokines, Journal of Ginseng Research. (2023) 47, no. 5, 627–637, 10.1016/j.jgr.2023.01.004. 37720571 PMC10499592

[31] Wang D. , Zhang H. , and Liao X. , et al.Oral Administration of *Robinia pseudoacacia* L. Flower Exosome-like Nanoparticles Attenuates Gastric and Small Intestinal Mucosal Ferroptosis Caused by Hypoxia Through Inhibiting Hif-1α- And Hif-2α-mediated Lipid Peroxidation, Journal of Nanobiotechnology. (2024) 22, no. 1, 10.1186/s12951-024-02663-6, 479. 39134988 PMC11321022

[32] Xu Y. , Huang C. , and Xu H. , et al.Modified Zhenwu Decoction Improved Intestinal Barrier Function of Experimental Colitis Through Activation of sGC-Mediated cGMP/PKG Signaling, Journal of Ethnopharmacology. (2024) 334, 10.1016/j.jep.2024.118570, 118570. 39002824

[33] Miao H. , Meng H. , Zhang Y. , Chen T. , Zhang L. , and Cheng W. , FSP1 Inhibition Enhances Olaparib Sensitivity in BRCA-Proficient Ovarian Cancer Patients via a Nonferroptosis Mechanism, Cell Death and Differentiation. (2024) 31, no. 4, 497–510, 10.1038/s41418-024-01263-z. 38374229 PMC11043371

[34] Zhang S. , Luo C. , and Li K. , et al.Baicalin Alleviates Intestinal Inflammation and Microbial Disturbances by Regulating Th17/Treg Balance and Enhancing Lactobacillus Colonization in Piglets, Journal of Animal Science and Biotechnology. (2024) 15, no. 1, 10.1186/s40104-024-01126-0, 172. 39707535 PMC11661242

[35] Sun Z. , Li J. , and Wang W. , et al.Qingchang Wenzhong Decoction Accelerates Intestinal Mucosal Healing Through Modulation of Dysregulated Gut Microbiome, Intestinal Barrier and Immune Responses in Mice, Frontiers in Pharmacology. (2021) 12, 10.3389/fphar.2021.738152, 738152. 34557102 PMC8452913

[36] Li H. , Pu X. , and Lin Y. , et al.Sijunzi Decoction Alleviates Inflammation and Intestinal Epithelial Barrier Damage and Modulates the Gut Microbiota in Ulcerative Colitis Mice, Frontiers in Pharmacology. (2024) 15, 10.3389/fphar.2024.1360972, 1360972. 38650625 PMC11033371

[37] Lee Y. S. , Kim T. Y. , and Kim Y. , et al.Microbiota-Derived Lactate Accelerates Intestinal Stem-Cell-Mediated Epithelial Development, Cell Host & Microbe. (2018) 24, no. 6, 833–846.e6, 10.1016/j.chom.2018.11.002, 2-s2.0-85057565842. 30543778

[38] Zhang L. , Tai Y. , and Zhao C. , et al.Inhibition of Cyclooxygenase-2 Enhanced Intestinal Epithelial Homeostasis via Suppressing Beta-Catenin Signalling Pathway in Experimental Liver Fibrosis, Journal of Cellular and Molecular Medicine. (2021) 25, no. 16, 7993–8005, 10.1111/jcmm.16730. 34145945 PMC8358882

[39] Buckley A. and Turner J. R. , Cell Biology of Tight Junction Barrier Regulation and Mucosal Disease, Cold Spring Harbor Perspectives in Biology. (2018) 10, no. 1. 10.1101/cshperspect.a029314 PMC5749156 28507021

[40] Huang S. , Wang X. , and Xie X. , et al.Dahuang Mudan Decoction Repairs Intestinal Barrier in Chronic Colitic Mice by Regulating the Function of ILC3, Journal of Ethnopharmacology. (2022) 299, 10.1016/j.jep.2022.115652, 115652. 36038092

[41] Sittipo P. , Shim J.-W. , and Lee Y. , Microbial Metabolites Determine Host Health and the Status of Some Diseases, International Journal of Molecular Sciences. (2019) 20, no. 21, 10.3390/ijms20215296, 2-s2.0-85074147329, 115652. PMC6862038 31653062

[42] Coelho N. R. , Pimpao A. B. , and Correia M. J. , et al.Pharmacological Blockage of the AHR-CYP1A1 Axis: A Call for In Vivo Evidence, Journal of Molecular Medicine. (2022) 100, no. 2, 215–243, 10.1007/s00109-021-02163-2. 34800164 PMC8605459

[43] Allan D. S. , Kirkham C. L. , and Aguilar O. A. , et al.An In Vitro Model of Innate Lymphoid Cell Function and Differentiation, Mucosal Immunology. (2015) 8, no. 2, 340–351, 10.1038/mi.2014.71, 2-s2.0-84922744283. 25138665

[44] Ye Q. , Huang S. , and Wang Y. , et al.Wogonin Improves Colitis by Activating the AhR Pathway to Regulate the Plasticity of ILC3/ILC1, Phytomedicine. (2024) 128, 10.1016/j.phymed.2024.155425, 155425. 38518634

[45] Li J. , Song J. , and Deng Z. , et al.Robust Reactive Oxygen Species Modulator Hitchhiking Yeast Microcapsules for Colitis Alleviation by Trilogically Intestinal Microenvironment Renovation, Bioactive Materials. (2024) 36, 203–220, 10.1016/j.bioactmat.2024.02.033. 38463553 PMC10924178

[46] Shen J. , Yang L. , and You K. , et al.Indole-3-Acetic Acid Alters Intestinal Microbiota and Alleviates Ankylosing Spondylitis in Mice, Frontiers in Immunology. (2022) 13, 10.3389/fimmu.2022.762580, 762580. 35185872 PMC8854167

[47] Dang G. , Wen X. , and Zhong R. , et al.Pectin Modulates Intestinal Immunity in a Pig Model via Regulating the Gut Microbiota-Derived Tryptophan Metabolite-AhR-IL22 Pathway, Journal of Animal Science and Biotechnology. (2023) 14, no. 1, 10.1186/s40104-023-00838-z, 38. 36882874 PMC9993796

[48] Long D. , Mao C. , Xu Y. , and Zhu Y. , The Emerging Role of Neutrophil Extracellular Traps in Ulcerative Colitis, Frontiers in Immunology. (2024) 15, 10.3389/fimmu.2024.1425251, 1425251. 39170617 PMC11335521

[49] Zhu H. C. , Jia X. K. , and Fan Y. , et al.Alisol B 23-Acetate Ameliorates Azoxymethane/Dextran Sodium Sulfate-Induced Male Murine Colitis-Associated Colorectal Cancer via Modulating the Composition of Gut Microbiota and Improving Intestinal Barrier, Frontiers in Cellular and Infection Microbiology. (2021) 11, 10.3389/fcimb.2021.640225, 640225. 33996624 PMC8117151

[50] Lee S. H. , Intestinal Permeability Regulation by Tight Junction: Implication on Inflammatory Bowel Diseases, Intestinal Research. (2015) 13, no. 1, 11–18, 10.5217/ir.2015.13.1.11. 25691839 PMC4316216

[51] Huang S. , Xie X. , and Xu B. , et al.Paeoniflorin Ameliorates Chronic Colitis via the DR3 Signaling Pathway in Group 3 Innate Lymphoid Cells, Journal of Pharmaceutical Analysis. (2024) 14, no. 6, 10.1016/j.jpha.2024.01.008, 100940. 39027912 PMC11255901

[52] Overcast G. R. , Meibers H. E. , and Eshleman E. M. , et al.IEC-Intrinsic IL-1R Signaling Holds Dual Roles in Regulating Intestinal Homeostasis and Inflammation, Journal of Experimental Medicine. (2023) 220, no. 6, 10.1084/jem.20212523. PMC10067527 36976181

[53] Schlegel N. , Boerner K. , and Waschke J. , Targeting Desmosomal Adhesion and Signalling for Intestinal Barrier Stabilization in Inflammatory Bowel Diseases-Lessons From Experimental Models and Patients, Acta Physiologica. (2021) 231, no. 1, 10.1111/apha.13492. 32419327

[54] Zhao M. , Xie X. , and Xu B. , et al.Paeonol Alleviates Ulcerative Colitis in Mice by Increasing Short-Chain Fatty Acids Derived From *Clostridium butyricum* , Phytomedicine. (2023) 120, 10.1016/j.phymed.2023.155056, 155056. 37703619

[55] Pickert G. , Neufert C. , and Leppkes M. , et al.STAT3 Links IL-22 Signaling in Intestinal Epithelial Cells to Mucosal Wound Healing, Journal of Experimental Medicine. (2009) 206, no. 7, 1465–1472, 10.1084/jem.20082683, 2-s2.0-67650474246. 19564350 PMC2715097

[56] Li Y. Y. , Wang X. J. , and Su Y. L. , et al.Baicalein Ameliorates Ulcerative Colitis by Improving Intestinal Epithelial Barrier via AhR/IL-22 Pathway in ILC3s, Acta Pharmacologica Sinica. (2022) 43, no. 6, 1495–1507, 10.1038/s41401-021-00781-7. 34671110 PMC9160000

[57] Zhang J. , Lin B. , and Zhang Y. , et al.Baitouweng Decoction Alleviates Ulcerative Colitis by Regulating Tryptophan Metabolism Through DOPA Decarboxylase Promotion, Frontiers in Pharmacology. (2024) 15, 10.3389/fphar.2024.1423307, 1423307. 38974042 PMC11224817

[58] Franzosa E. A. , Sirota-Madi A. , and Avila-Pacheco J. , et al.Gut Microbiome Structure and Metabolic Activity in Inflammatory Bowel Disease, Nature Microbiology. (2019) 4, no. 2, 293–305, 10.1038/s41564-018-0306-4, 2-s2.0-85058195128. PMC6342642 30531976

[59] Zhu Y. , Huang X. , and Deng Z. , et al.Orally Biomimetic Metal-Phenolic Nanozyme with Quadruple Safeguards for Intestinal Homeostasis to Ameliorate Ulcerative Colitis, Journal of Nanobiotechnology. (2024) 22, no. 1, 10.1186/s12951-024-02802-z, 545. 39238009 PMC11378530

[60] Wang J. , Zhu N. , Su X. , Gao Y. , and Yang R. , Gut-Microbiota-Derived Metabolites Maintain Gut and Systemic Immune Homeostasis, Cells. (2023) 12, no. 5, 10.3390/cells12050793, 793. 36899929 PMC10000530

[61] AbdelMassih A. , Yacoub E. , and Husseiny R. J. , et al.Hypoxia-Inducible Factor (HIF): The Link Between Obesity and COVID-19, Obesity Medicine. (2021) 22, 10.1016/j.obmed.2020.100317, 100317. 33521378 PMC7832240

[62] Lin H.-T. , Cheng M.-L. , Lo C.-J. , Lin G. , and Liu F.-C. , Metabolomic Signature of Diabetic Kidney Disease in Cerebrospinal Fluid and Plasma of Patients With Type 2 Diabetes Using Liquid Chromatography-Mass Spectrometry, Diagnostics. (2022) 12, no. 11, 10.3390/diagnostics12112626, 2626. 36359470 PMC9689120

[63] Zhen J. , Zhao P. , and Li Y. , et al.The Multiomics Analyses of Gut Microbiota, Urine Metabolome and Plasma Proteome Revealed Significant Changes in Allergy Featured With Indole Derivatives of Tryptophan, Journal of Asthma and Allergy. (2022) 15, 117–131, 10.2147/JAA.S334752. 35125876 PMC8809677

[64] Zhang S. , Xia J. , Zhu Y. , Dong M. , and Wang J. , Establishing Salvia Miltiorrhiza-Derived Exosome-Like Nanoparticles and Elucidating Their Role in Angiogenesis, Molecules. (2024) 29, no. 7, 10.3390/molecules29071599, 1599. 38611878 PMC11013048

[65] Agus A. , Planchais J. , and Sokol H. , Gut Microbiota Regulation of Tryptophan Metabolism in Health and Disease, Cell Host & Microbe. (2018) 23, no. 6, 716–724, 10.1016/j.chom.2018.05.003, 2-s2.0-85047213592. 29902437

[66] Li S. , Modulation of Immunity by Tryptophan Microbial Metabolites, Frontiers in Nutrition. (2023) 10, 10.3389/fnut.2023.1209613, 1209613. 37521424 PMC10382180

